# Integrated transcriptomic and metabolomic profiling reveals coordinated regulatory networks associated with mosaic disease resistance in sugarcane

**DOI:** 10.3389/fpls.2026.1891189

**Published:** 2026-07-20

**Authors:** Mingjie You, Hui Li, Yan Xie, Ze Liu, Xuelu Wei, Jiahe Wu, Yi Xu, Qing Zhang, Jisen Zhang, Qiutao Xu, Heyang Shang

**Affiliations:** 1 Guangxi Sugarcane Bio-breeding Laboratory, State Key Laboratory for Conservation and Utilization of Subtropical Agro-bioresources, College of Agriculture, Guangxi University, Nanning, Guangxi, China; 2 Guangxi Key Lab of Agricultural Resources Chemistry and Biotechnology, Yulin Normal University, Yulin, China; 3 College of Life Science and Technology, Guangxi University, Nanning, Guangxi, China

**Keywords:** autophagy, cultivar-specific resistance, multi-omics integration, peroxisome homeostasis, sugarcane mosaic disease

## Abstract

**Introduction:**

Sugarcane mosaic disease (SCMD) poses a severe threat to global sugarcane yield. Since conventional field management is insufficient to restrict viral transmission, unraveling the underlying defense mechanisms is imperative for targeted breeding. The primary objective of this study was to delineate the molecular and metabolic networks governing SCMD resistance by comparing highly resistant (XIDAZHE10-19, YT94-128) and susceptible (HP, XTT22) cultivars.

**Methods:**

We employed metabolomic profiling and integrated transcriptomic data to investigate the genetic basis driving host responses. To further elucidate the functional and regulatory mechanics of identified key hub genes, we conducted weighted gene co-expression network analysis (WGCNA) alongside AlphaFold-driven structural predictions and interactome profiling.

**Results:**

Metabolomic profiling identified critical defense-associated metabolites --including alcoholamines and glycerol derivatives --that strongly correlate with disease incidence. Transcriptomic integration yielded two major findings. First, pathway enrichment revealed a striking dichotomy in defense strategies: XIDAZHE10-19 preferentially orchestrated the autophagy pathway and aromatic amino acid biosynthesis, whereas YT94-128 relied heavily on calcium signaling and peroxisome-mediated reactive oxygen species (ROS) homeostasis. Second, WGCNA pinpointed *ShNDK* as a core hub gene exhibiting robust upregulation in susceptible cultivars. AlphaFold predictions further revealed that *ShNDK* potentially assembles into dimers and physically associates with canonical immune transcription factors (e.g., bZIP, Dof) and pathogenesis-related (PR) proteins.

**Discussion:**

The novelty of this work lies in uncovering divergent, cultivar-specific defense strategies and identifying novel genetic hubs through a multi-omics and structural biology approach. Together, these findings unveil a complex, multi-layered defense network against SCMD. The characterization of *ShNDK* and the elucidation of cultivar-specific synergistic crosstalk provide a crucial mechanistic foundation and promising genetic targets for developing sugarcane cultivars with heritable, broad-spectrum resistance.

## 1 Introduction

Sugarcane is the most vital sugar crop, contributing more than 70% of global sugar production and over 90% of the total sugar output in China (Li and Yang, 2015).Characterized by high biomass and fiber content, sugarcane also serves as a critical source of renewable bioenergy (Waclawovsky et al., 2010). Sugarcane mosaic disease (SCMD) is one of the primary diseases affecting this crop; it severely restricts both yield and quality, thereby hindering the healthy and sustainable development of the sugarcane industry (Lu et al., 2021).In China, sugarcane cultivation regions—including Guangxi, Hainan, Yunnan, Guizhou, Sichuan, Guangdong, Fujian, and Zhejiang—have all been affected by sugarcane mosaic virus infections. When the incidence of sugarcane mosaic disease reaches 75%, yield losses range from 5% to 19%. In the upland sugarcane areas of southern China, the incidence exceeds 30%, while in highly susceptible varieties, it can reach 100%. Such infections lead to a 10.6% to 35.3% reduction in seed germination rates, yield losses between 3% and 50%, shortened internodes, and overall quality degradation (Rice and Hoy, 2020; Rice et al., 2019).

The primary pathogens of sugarcane mosaic disease belong to the family Potyviridae, specifically Sugarcane mosaic virus (SCMV), Sorghum mosaic virus (SrMV), and Sugarcane streak mosaic virus (SCSMV) (Lu et al., 2021).The viral species infecting different sugarcane-growing regions exhibit distinct variations. Sorghum mosaic virus (SrMV) is the predominant pathogen in regions including Guangxi, Guangdong, Fujian, Sichuan, Guizhou, and Yunnan; whereas Sugarcane mosaic virus (SCMV) is the primary species in Zhejiang, and Sugarcane streak mosaic virus (SCSMV) prevails in Hainan. Previous studies have demonstrated that the detection rates of these viruses in Guangxi, Fujian, and Yunnan follow the hierarchy of SrMV > SCSMV > SCMV, with the prevalence of SrMV being significantly higher than that of the other two. Furthermore, the rate of mixed infections (co-infections) is notably higher compared to that of single-virus infections (D-L et al., 2008; He et al., 2022).Due to the high spatiotemporal heterogeneity of viral species across different regions and cultivars, the prevention and control of this disease are extremely challenging and often yield suboptimal results (Lu et al., 2021).

Furthermore, the widespread presence and difficult eradication of alternative field hosts for sugarcane mosaic disease pathogens make it nearly impossible to completely sever the viral transmission chain. Consequently, conventional strategies—such as utilizing virus-free seedlings, disinfecting agricultural tools, and controlling insect vectors— may not fully eliminate. Breeding and deploying resistant varieties remain the most economical, effective, and environmentally safe measures for sugarcane mosaic disease management. However, most current resistant cultivars target only a single virus, exhibiting insufficient broad-spectrum resistance to mixed infections. Given that traditional cross-breeding is a protracted process with long cycles, it struggles to meet the urgent demand for resistant varieties. In this context, molecular breeding offers a rapid and efficient pathway for the selection and development of resistant sugarcane cultivars (Dal-Bianco et al., 2012; Lu et al., 2021; Moose and Mumm, 2008). Currently, the predominant approach in molecular breeding for sugarcane mosaic disease resistance involves integrating the viral coat protein (CP) gene into the sugarcane genome to generate transgenic plants, followed by validation through artificial inoculation and field resistance assessments. However, CP-mediated resistance is highly contingent upon sequence homology. The emergence of heterologous viral strains with low homology poses a significant risk, as modern sugarcane cultivars may lose their resistance, potentially triggering large-scale sugarcane mosaic disease epidemics (Ingelbrecht et al., 1999). Complementary to genetic resistance, synthetic antiviral agents have also been explored; for instance, morpholine ring-containing pyrazoline acylhydrazone derivatives exhibit potent activity against Tobacco mosaic virus (TMV) through mechanisms distinct from CP-mediated resistance (Liao et al., 2026). While these previous findings have successfully demonstrated targeted viral suppression, a critical knowledge gap persists regarding the plant’s endogenous molecular mechanisms for broad-spectrum resistance. Specifically, the functional resistance genes and the systems-level regulatory networks that govern sugarcane immunity against multi-virus infection complexity are still largely unknown. To bridge this gap and overcome the limitations of narrow-spectrum resistance, the identification of broad-spectrum resistance genes is imperative. However, traditional single-omics studies often fail to capture the holistic nature of plant immunity. Therefore, we aim to employ a novel integrated multi-omics strategy combining transcriptomics and metabolomics. This integration uniquely bridges the gap between gene transcription and biochemical phenotypic responses, providing a comprehensive view of the defense cascade (Li et al., 2020). The primary objective of this study is to systematically decode the global regulatory networks underlying SCMD resistance and to identify core functional hub genes. We hypothesize that resistant cultivars employ distinct, pathway-specific defense strategies that can be dissected by integrated multi-omics. Ultimately, elucidating these systems-level mechanisms will provide robust genetic targets and a crucial theoretical foundation, thereby accelerating molecular breeding strategies for developing sugarcane cultivars with heritable, broad-spectrum resistance.

## 2 Materials and methods

### 2.1 Plant materials

From 2023 to 2025, an investigation into the incidence of sugarcane mosaic disease was conducted at the Guangxi Subtropical Agricultural Science and Technology New City Experimental Base in Fusui. This study utilized two resistant cultivars (lines) and two susceptible cultivars, previously screened by our research group, as research materials. Specifically, the study evaluated the known resistant cultivars XIDAZHE10-19 and YT94-128 alongside the susceptible cultivars Huangpiguozhe (HP) and XTT22. To ensure statistical robustness, the field trial was established using a randomized complete block design (RCBD) with three independent replications. Within each block, the four cultivars were randomly assigned to individual experimental plots to minimize potential spatial bias and environmental confounding effects. Each plot featured a row length of 5 meters and a row spacing of 1.2 meters, with a 1-meter interval maintained between different materials to prevent cross-interference. Guard rows were planted around the perimeter of the entire experimental area to minimize edge effects. The planting density was standardized at 12 buds per meter across all plots. During the field investigation, disease incidence was recorded and calculated for each plot using the following formula: Disease Incidence (%) = (Number of plants exhibiting mosaic symptoms/Total number of plants surveyed) × 100 (Li et al., 2014). Leaf tissue samples were collected from each cultivar on April 21, 2025. Upon collection, the samples were immediately flash-frozen in liquid nitrogen and subsequently stored in a -80 °C freezer for further transcriptomic and metabolomic sequencing.

### 2.2 Detection of the virus

Given its high efficiency, rapid turnaround, cost-effectiveness, and ease of operation, standard PCR was selected for the qualitative detection of the viruses in this study (Wang et al., 2019). PCR amplification of the target viral fragments was performed using Taq DNA Polymerase, utilizing cDNA reverse-transcribed from the total RNA of the transcriptomics samples as the template. The PCR reaction mixture (total volume of 20 μL) consisted of 10 μL of 2× Taq Master Mix, 0.8 μL of forward primer (10 μM), 0.8 μL of reverse primer (10 μM), 1.0 μL of cDNA template, and sterile ddH_2_O to a final volume of 20 μL. The specific primers utilized in this study are listed in **Supplementary Tables S1**-1. For the assay controls, total nucleic acids extracted from sugarcane leaves infected with SCSMV, SCMV, and SrMV were used as the positive control, while samples from virus-free sugarcane seedlings served as the negative control.

The amplification was conducted in a thermal cycler under the following conditions: initial denaturation at 95 °C for 5 min; followed by 35 cycles of denaturation at 95 °C for 30 s, annealing at 58 °C for 30 s, and extension at 72 °C for 45 s; with a final extension step at 72 °C for 10 min, and then held at 4 °C. The resulting PCR products were resolved by electrophoresis on a 1.0% agarose gel and visualized using a gel imaging system.

### 2.3 Transcriptome sequencing and analysis

Samples were transported on dry ice to Beijing Berry Genomics Co., Ltd. for transcriptome sequencing. Only samples with an RNA Integrity Number (RIN) ≥ 7 were subjected to subsequent analysis. Raw sequencing data (Raw reads) were filtered using Trimmomatic software to obtain high-quality clean reads (Bolger et al., 2014). Subsequently, the clean reads were aligned to the XTT22 reference genome using HISAT2 (version 2.1.0) (Kim et al., 2019; Zhang et al., 2025), and the number of reads mapped to each gene was quantified using featureCounts (version 2.0.2) (Liao et al., 2014). Differentially expressed genes (DEGs) were identified using the DESeq2 R package (version 1.40.2), with screening thresholds set at |log2 FC| > 1 and *p* < 0.05 (Love et al., 2014). Finally, based on the identified DEGs, Gene Ontology (Ashburner et al., 2000) and Kyoto Encyclopedia of Genes and Genomes (KEGG) (Ogata et al., 1999) pathway enrichment analyses were performed using a hypergeometric test. This analysis aimed to identify pathways significantly enriched relative to the genomic background (significance threshold set at *p* < 0.05), The top 20 pathways with the lowest p-values were selected for visualization.

### 2.4 Weighted gene co-expression network analysis

Weighted gene co-expression network analysis was performed using the WGCNA R package (Langfelder and Horvath, 2008). The expression data were first preprocessed by filtering out genes and samples with zero variance or missing values using the goodSamplesGenes function to ensure data quality. To ensure the constructed network adhered to the scale-free topology characteristics of biological systems (Barabási and Albert, 1999), an appropriate soft-thresholding power was selected. Given that a scale-free fit index (R²) between 0.8 and 0.9 is generally considered sufficient to indicate that a network satisfies the scale-free topology criterion, an R² threshold of 0.85 was applied in this study (Langfelder and Horvath, 2008). Subsequently, an unsigned co-expression network was constructed, and the adjacency matrix was transformed into a topological overlap matrix (TOM). Genes were hierarchically clustered based on 1-TOM dissimilarity, and co-expression modules were partitioned using the dynamic tree cut algorithm (minimum module size = 30). Highly similar modules were merged using a cut height (mergeCutHeight) of 0.25. To identify modules associated with phenotypic traits, Pearson correlation coefficients were calculated between module eigengenes (MEs) and trait data. Specifically, the disease incidence was analyzed as a continuous variable, whereas the symptom manifestation was analyzed as a categorical variable. Within the most significantly trait-related modules, hub genes were identified based on stringent criteria: absolute module membership (|MM| > 0.8) and absolute gene significance (|GS| > 0.5). Finally, the hub genes and their topological interactions (edge weight threshold > 0.1) were exported and visualized using Cytoscape software (Shannon et al., 2003).

### 2.5 Metabolite extraction and analysis

Non-targeted metabolomics was performed by Wuhan Maiwei Metabolic Biotechnology. Extracts were analyzed in both positive and negative ionization modes using a Shimadzu LC-30A UPLC coupled to a Triple TOF 6600+ mass spectrometer. Chromatographic separation utilized a Waters ACQUITY UPLC HSS T3 column (1.8 µm, 2.1 × 100 mm) at 40 °C, with a 0.40 mL/min flow rate of 0.1% formic acid in water (A) and acetonitrile (B). Quality control (QC) samples, pooled from equal aliquots of all biological samples, were injected every 10 samples to monitor system stability. Raw data were converted to mzML (ProteoWizard) and processed via XCMS for peak extraction, alignment, and retention time correction. After filtering peaks with >50% missing values per group, missing data were imputed (KNN or 1/5 minimum) and peak areas were SVR-corrected. Metabolites were annotated utilizing integrated public and proprietary databases. High-quality compounds (comprehensive score ≥ 0.5, QC CV < 0.5) from both ionization modes were merged. Significant differential metabolites were defined by an OPLS-DA variable importance in projection (VIP) score > 1 and a fold change (FC) ≥ or ≤ 0.5. Volcano plots were utilized to visualize the overall distribution and the counts of up- and down-regulated DEMs for each comparison group. Finally, the collective set of identified DEMs was subjected to KEGG pathway enrichment analysis (Ogata et al., 1999), using their respective KEGG IDs. A hypergeometric test was applied to identify pathways significantly enriched relative to the metabolic background (p < 0.05).

### 2.6 Multi-omics integration and correlation analysis

Network-based integration was performed to map differentially expressed genes (DEGs) and differentially expressed metabolites (DEMs) onto concurrent KEGG pathways. To construct transcriptomic co-expression modules, Weighted Gene Co-expression Network Analysis (WGCNA) was utilized. Subsequently, Pearson correlation analysis was conducted between the WGCNA module eigengenes and the abundance of key DEMs to establish module-metabolite correlations and extract core regulatory subnetworks.

### 2.7 RNA extraction and quantitative real-time PCR

To experimentally validate the transcript expression profiles of the computationally identified hub genes, total RNA was extracted and used for cDNA synthesis with the StarScript II First Strand cDNA Synthesis Kit (with gDNA Removal Agent) (A222-10, Genstar). Quantitative real-time PCR (RT-qPCR) amplification was performed on a Bio-Rad fluorescence detection system using the 2× RealStar Green Fast Mixture (A301-10, Genstar). The specific primers utilized for RT-qPCR are listed in **Supplementary Table 8**. The reaction followed a two-step protocol: an initial denaturation at 95 °C for 2 min, followed by 40 cycles of denaturation at 95 °C for 15 s and annealing/extension at 60 °C for 30 s. Gene expression levels were quantified using the 2−ΔΔCt method (Livak and Schmittgen, 2001), with GAPDH as the reference gene (Iskandar et al., 2004; Yang et al., 2016). Data visualization was conducted using GraphPad Prism 9.0.

### 2.8 Protein-protein interaction and 3D structure prediction

Protein-protein interaction (PPI) predictions were performed based on *Saccharum* hybrid cultivar XTT22 and *Saccharum spontaneum* SES208 protein sequence using AlphaFold (v4.0) (Evans et al., 2021). To ensure the reliability of the predicted PPI complex structures, rigorous quality control was implemented by integrating multiple structural evaluation metrics. Based on established standards (Genz et al., 2025), a C2Q score ≥ 0.52 was employed as the threshold for screening high-confidence interaction models. Additionally, three-dimensional (3D) structural modeling of key proteins was conducted via the SWISS-MODEL platform (https://swissmodel.expasy.org) (Waterhouse et al., 2018). Ab initio molecular docking of the target proteins was carried out using the HDOCK server (http://hdock.phys.hust.edu.cn/) (Yan et al., 2020). Notably, all PPI networks, structural topologies, and docking complexes presented in this section represent computationally predicted interactions (in silico), rather than experimentally validated physical interactions.

## 3 Result

### 3.1 Incidence of sugarcane mosaic disease in four sugarcane cultivars

**Figure 1:**
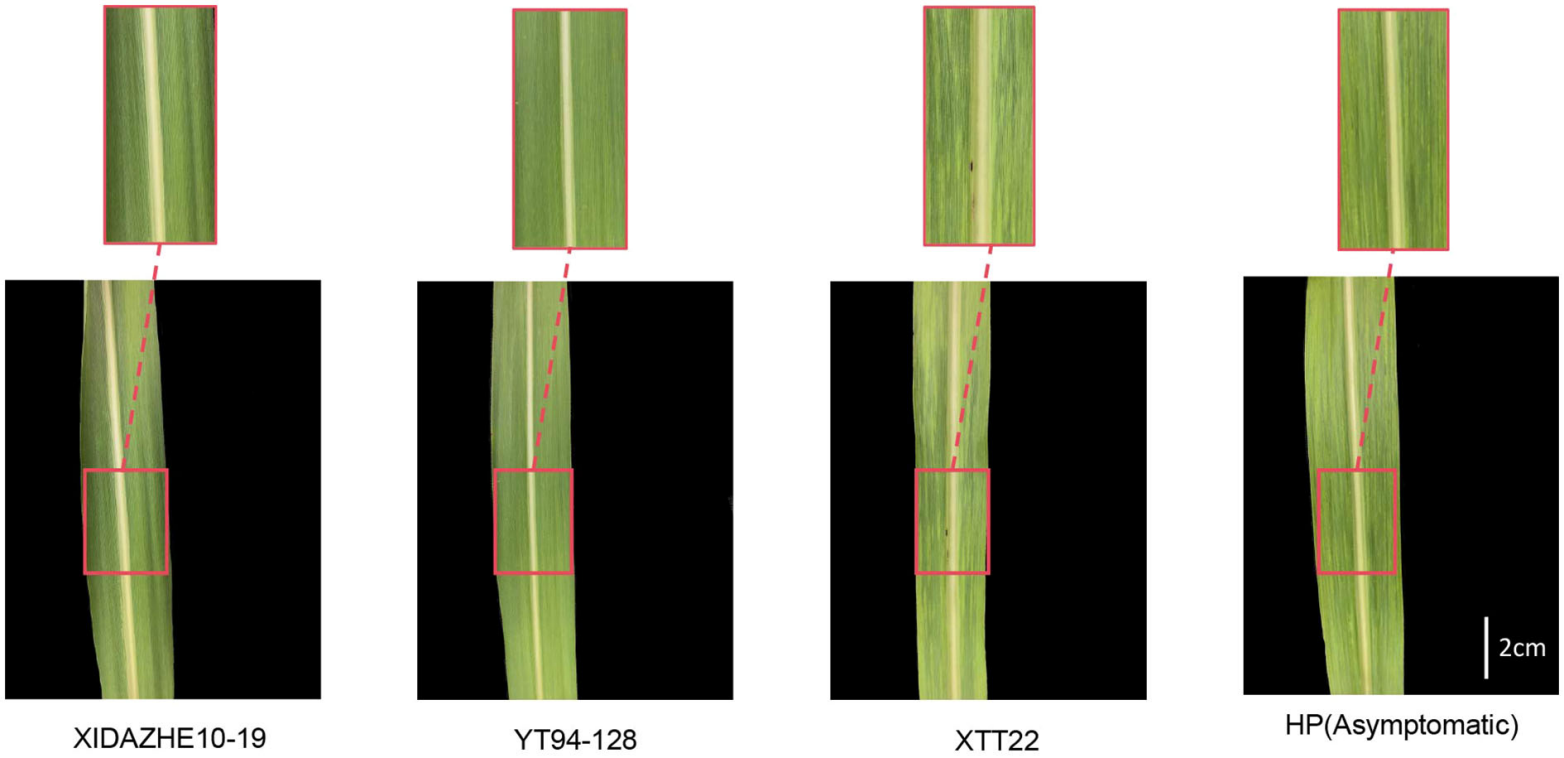
Phenotypic characterization of sugarcane cultivars in response to mosaic virus infection. Leaf phenotypes of resistant and susceptible cultivars. Red dashed boxes represent close-up views of the indicated are as (Scale bar = 2 cm). Top row: Resistant cultivars (XIDAZHE10-19 and YT94-128) display healthy green leaves without obvious chlorosis. Bottom row: Susceptible cultivars (XTT22 and HP) exhibit severe mosaic symptoms characterized by irregular chlorotic streaks and patches.

A multi-year field investigation (2023–2025) was conducted to systematically monitor the incidence of sugarcane mosaic disease. Over the three-year observation period, year-wise trends revealed distinct phenotypic responses among the tested cultivars. Specifically, the highly susceptible cultivar HP maintained a consistent 100% disease incidence across all three consecutive years (2023, 2024, and 2025), regardless of whether it was newly planted or managed as ratoon cane. In contrast, the incidence in cultivar XTT22 exhibited significant temporal fluctuation depending on the propagation source; it reached a 100% incidence rate only when propagated directly from diseased canes from the previous year, whereas its incidence decreased significantly in conventional newly planted and ratoon crops. This variable incidence strongly implies that XTT22 is highly vulnerable to seed-borne virus transmission, highlighting the critical risks of latent viral infections accumulating during vegetative propagation. Regarding XIDAZHE10-19 and YT94-128, no visual disease symptoms (0% incidence) were observed in either newly planted or ratoon crops across all surveyed years (**Figure 1**; **Supplementary Table 1**). These results further corroborate the identification of these resistant and susceptible materials reported in previous studies. To further determine the underlying viral species infecting these four cultivars, Polymerase Chain Reaction (PCR) assays were performed (primer information is provided in **Supplementary Table 1**). The results demonstrated a high prevalence of mixed infections: the asymptomatic resistant cultivars YT94-128 and XIDAZHE10-19 were co-infected with SCMV and SCSMV, whereas the symptomatic cultivars HP and XTT22 were co-infected with SCMV and SrMV (**Supplementary Tables 1**–**3**). The stark contrast between the phenotypic disease incidence and these complex mixed-infection profiles underscores the diverse defense strategies of the host plants. Consequently, these specific cultivars were selected as ideal models for our subsequent molecular and multi-omics analyses, aiming to explicitly dissect the mechanisms underlying broad-spectrum resistance to mixed viral infections.

### 3.2 Transcriptomic analysis of resistant and susceptible sugarcane cultivars against sugarcane mosaic disease

#### 3.2.1 Global transcriptomic profiles and identification of differentially expressed genes among different sugarcane cultivars

**Figure 2:**
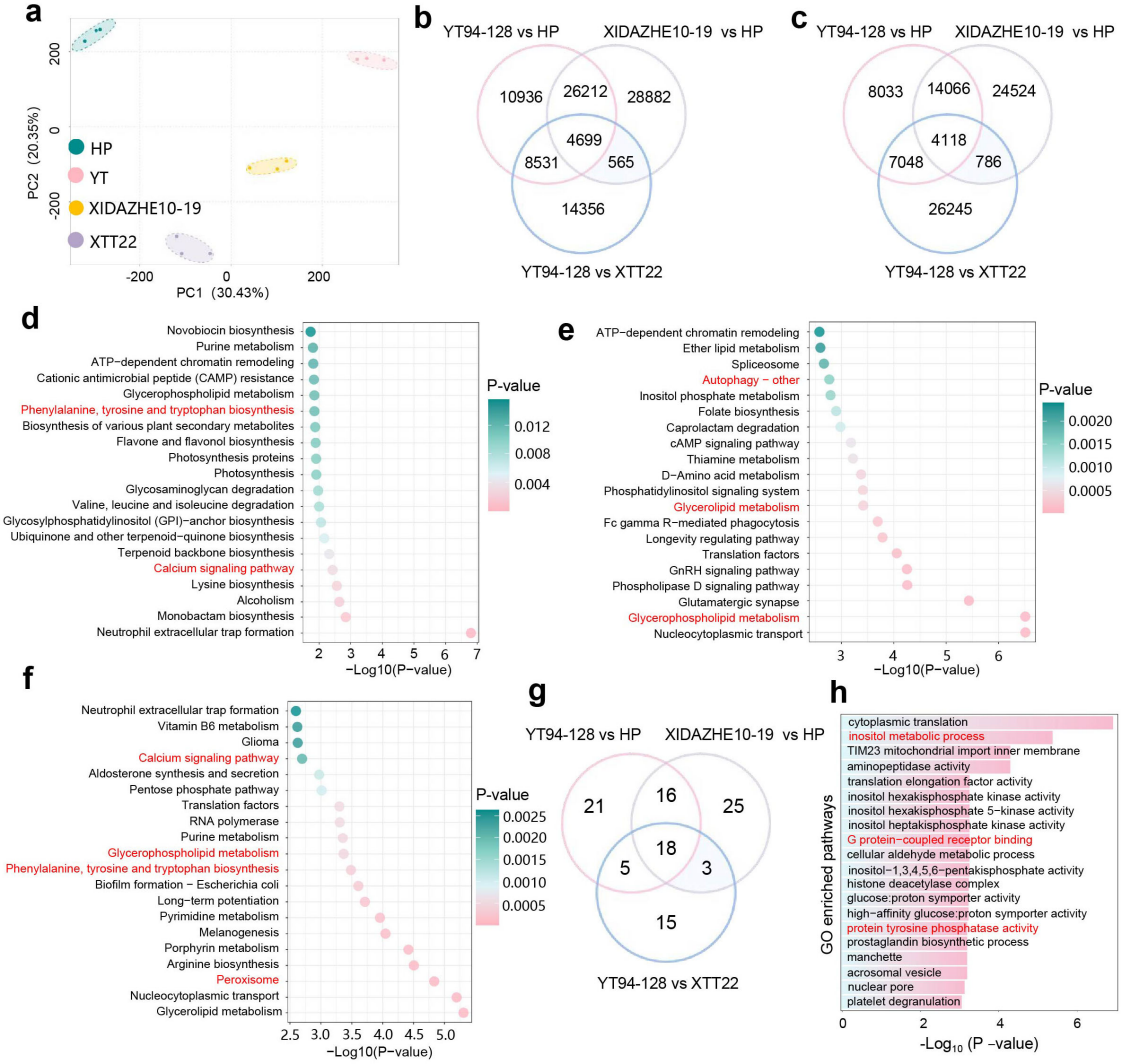
Transcriptomic and functional enrichment analyses of differentially expressed genes (DEGs) between resistant and susceptible sugarcane cultivars. **(a)** Principal component analysis (PCA) of transcriptomic data from the four sugarcane cultivars (HP, YT94-128, XIDAZHE10-19, and XTT22), illustrating the clustering among samples. **(b, c)** Venn diagrams showing the overlap of unique and shared up-regulated **(b)** and down-regulated **(d)** DEGs across three comparison groups (YT94-128 vs. HP, XIDAZHE10-19 vs. HP, and YT94-128 vs. XTT22).(e–g) KEGG pathway enrichment analysis for DEGs in **(e)** YT94-128 vs. HP, **(f)** XIDAZHE10-19 vs. HP, and **(g)** YT94-128 vs. XTT22. **(h)** Venn diagram displaying the intersection of significantly enriched KEGG pathways among the three comparison groups. **(h)** Bar chart of Gene Ontology (GO) enrichment analysis for DEGs in the YT94-128 vs HP comparison group.

To dissect the molecular mechanisms underlying sugarcane responses to sugarcane mosaic disease, transcriptomic analyses were comprehensively performed on the cultivars HP, XTT22, XIDAZHE10-19, and YT94-128. Correlation analysis among biological replicates revealed high correlation coefficients, demonstrating the robust reproducibility and reliability of the RNA-seq data (**Supplementary Figure 1b**). To further evaluate the overall variation in gene expression patterns among the cultivars, principal component analysis (PCA) was conducted based on transcript abundance. The PCA results showed clear separation among the four cultivars, with principal components 1 (PC1) and 2 (PC2) explaining 30.43% and 20.35% of the total variance, respectively, indicating substantial differences in their global gene expression profiles (**Figure 2a**). Volcano plots were constructed to visualize the different expressed genes (DEGs) (|log2 FC| > 1 and p < 0.05) across various comparison groups. In the YT94-128 vs. HP comparison group, a total of 83,642 DEGs were identified, comprising 50,380 down-regulated and 33,262 up-regulated DEGs (**Supplementary Figure 1b**). The XIDAZHE10-19 vs. HP comparison revealed 103,597 DEGs, including 60,175 down-regulated and 43,422 up-regulated DEGs (**Supplementary Figure 1c**). In the YT94-128 vs. XTT22 group, 66,342 DEGs were detected, with 28,151 down-regulated and 38,191 up-regulated genes (**Supplementary Figure 1d**). Finally, Venn diagrams for up- and down-regulated DEGs demonstrated that 4,118 up-regulated DEGs and 4,699 down-regulated DEGs were collectively shared among these three comparison groups (**Figures 2b, c**; **Supplementary Table 2**). To establish a robust foundation for downstream analyses, we explicitly prioritized this core set of 8,817 shared DEGs, as they represent the most conserved, genotype-independent transcriptional responses to SCMD.

To systematically characterize the biological roles of these shared up- and down-regulated DEGs, functional categorization was initially performed. As will be systematically detailed in the subsequent section, the results revealed that these common DEGs were predominantly enriched in critical biological processes and signaling networks, particularly including signal transduction (such as the calcium signaling pathway and G protein-coupled receptor binding), lipid/inositol metabolism, and specific amino acid biosynthesis (e.g., phenylalanine, tyrosine and tryptophan biosynthesis). Subsequently, hierarchical clustering was performed on these shared up- and down-regulated DEGs to generate an expression heatmap (**Supplementary Figure 1e**). By integrating these expression profiles with sugarcane mosaic disease (SCMD) incidence rates, genes highly correlated with disease development were identified from these key functional categories. For instance, *Sh_So01J0081988*, which encodes a photosystem I reaction center subunit III, exhibited notably high expression levels in the susceptible cultivars (HP and XTT22). Given that plant viruses frequently hijack chloroplast machinery and host energy reserves for their own replication, the sustained upregulation of this photosynthetic gene suggests that susceptible cultivars may fail to execute a timely growth-to-defense transition, thereby inadvertently supplying abundant resources for viral proliferation. Additionally, we identified *Sh_Ss04H0210788*, which encodes a light-harvesting complex (LHC) protein functioning as a photoreceptor. This gene displayed relatively high expression in susceptible cultivars and was significantly enriched in disease resistance-related pathways, such as phenylalanine metabolism. Its paradoxically high expression in susceptible plants implies a potential viral manipulation of host light signaling. Such manipulation likely disrupts the normal metabolic flux of the phenylalanine pathway, impairing the timely synthesis of downstream defensive secondary metabolites (e.g., lignin and flavonoids) and ultimately exacerbating disease susceptibility.

#### 3.2.2 Functional and pathway enrichment analyses of DEGs across different comparison groups

To systematically characterize the pathway enrichment profiles of DEGs across different comparison groups, the KEGG pathway enrichment analysis for the DEGs was performed (**Supplementary Table 3**; **Figures 2e–g**). The results showed that the top 20 significantly enriched pathways for the comparison groups XIDAZHE10-19 vs. HP, YT94-128 vs. XTT22, and YT94-128 vs. HP. In the XIDAZHE10-19 vs. HP comparison group, key pathways associated with disease resistance primarily included the calcium signaling pathway and PPAR signaling pathway. In the YT94-128 vs. XTT22 comparison group, key resistance pathways encompassed terpenoid backbone biosynthesis, anthocyanin biosynthesis, and fatty acid elongation. Furthermore, for the YT94-128 vs. HP comparison group, the significantly enriched key pathways related to disease resistance included phenylalanine, tyrosine and tryptophan biosynthesis, autophagy - other, endocytosis, phospholipase D signaling pathway and glutathione metabolism (Lefevere et al., 2020; Wang et al., 2025b).

A Venn diagram was generated based on the significantly enriched KEGG pathways of the DEGs. The intersection revealed that 18 significantly enriched pathways were shared across the three comparison groups. These included several disease resistance-related pathways, such as the calcium signaling pathway, necroptosis, GTP-binding proteins, autophagy - other, and peroxisome (**Figure 2h**, **Supplementary Table 3**) (Cai et al., 2022; Huang et al., 2022).

To further comprehensively characterize the functional attributes, molecular activities, and cellular localization of these DEGs, Gene Ontology (GO) enrichment analysis was performed (**Supplementary Table 4**). According to the analysis, the disease resistance-related terms enriched in the XIDAZHE10-19 vs. HP comparison group included cysteine-type endopeptidase inhibitor activity, regulation of cytokine-mediated signaling pathway, and peroxisomal membrane (**Supplementary Figure 1f**). In the YT94-128 vs. HP comparison group, the enriched resistance-associated terms comprised mannose-1-phosphate guanylyltransferase activity, protein peptidyl-prolyl isomerization, and nitrilase activity (**Figure 2h**). Furthermore, in the YT94-128 vs. XTT22 comparison group, the enriched resistance-related GO terms encompassed the THO complex, MAP kinase activity, calmodulin-dependent protein kinase activity, and phospholipase D activity (**Supplementary Figure 1g**) (Calero-Muñoz et al., 2019; Demers et al., 2023; Kim et al., 2026; Wilhelm et al., 2022).

#### 3.2.3 Expression analysis of DEGs in calcium signaling and autophagy pathways

**Figure 3:**
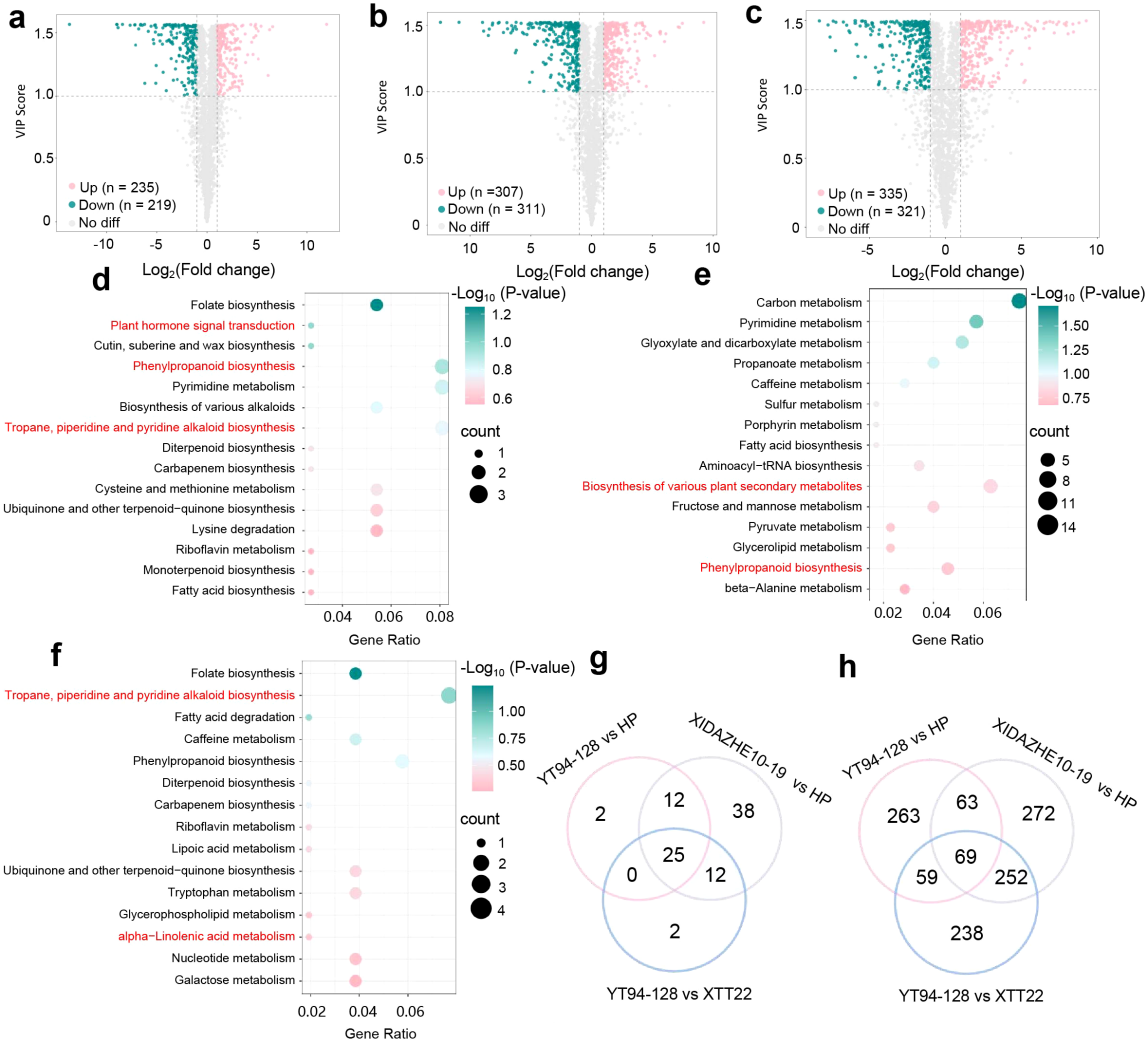
Identification and functional enrichment analysis of differentially accumulated metabolites (DAMs) between resistant and susceptible sugarcane varieties. **(a–c)** Volcano plots of DAMs in the comparison groups YT94-128 vs. XTT22 **(a)**, YT94-128 vs. HP **(b)**, and XIDAZHE10-19 vs. HP **(c)**. The x-axis represents the-Log2(Fold Change), and the y-axis represents the VIP value. **(d–f)** KEGG pathway enrichment scatter plots of all DAMs in the comparison groups YT94-128 vs. XTT22 **(d)**, YT94-128 vs. HP. **(e)**, and XIDAZHE10-19 vs. HP **(f)**. The size of the circles indicates the number of metabolites enriched in each pathway, and the color gradient represents the significance level of the P-value. **(g)** Venn diagram showing the intersection of significantly enriched KEGG pathways among the three comparison groups. **(h)** Venn diagram showing the overlap of unique and shared DAMs among the three comparison groups.

To pinpoint the distinct defense mechanisms employed by different sugarcane cultivars, we focused our attention on the calcium signaling and autophagy pathways prioritized from the shared KEGG categories. By characterizing the expression dynamics of the constituent genes within these two pathways across the four cultivars, we aimed to delineate the specific disease resistance strategies predominantly relied upon by each cultivar during viral invasion. Calcium ions Ca^2+^, acting as crucial second messengers, play a vital role in signal transduction during plant disease resistance. During the early stages of viral infection, a rapid and robust Ca^2+^ burst occurs within plant cells, and the transmembrane flux of these Ca^2+^ signals effectively activates downstream defense cascades (Lederer et al., 2025; Li et al., 2026). The identification and expression analysis of DEGs in the calcium signaling pathway revealed differential expression of core genes among the four sugarcane cultivars (**Figures 3a**; **Supplementary Table 7**). For instance, *Sh_Ss04G0209898*, encoding a calmodulin-dependent protein kinase 2 (CAMK2), was highly expressed in YT94-128. Conversely, genes such as *Sh_Ss09G0329149*, which encode calcineurin (CaN), exhibited relatively high expression levels in XTT22 and XIDAZHE10-19 (**Supplementary Table 7**) (Deng et al., 2026).

Autophagy serves as a critical defense mechanism for plants against viral invasion. By driving the assembly of autophagosomes, which subsequently engulf and degrade viral proteins or particles, it significantly reduces the intracellular viral load (Wang et al., 2025a; Yang et al., 2025). As illustrated by the enrichment of autophagy-related DEGs in the autophagy pathway (**Figure 3b**), genes such as *Sh_So02F0117619* and *Sh_So07K0292726*—which encode ATG8, a hallmark protein essential for autophagosome formation—exhibited higher expression levels in XTT22, XIDAZHE10-19, and YT94-128, suggesting these cultivars may rely more heavily on autophagy, but relatively lower expression in HP. In contrast, *Sh_So04A0187358*, which encodes the autophagy cofactor ATG101, showed notably high expression specifically in HP.

### 3.3 Metabolomic profiling and pathway enrichment of differentially expressed metabolites

**Figure 4:**
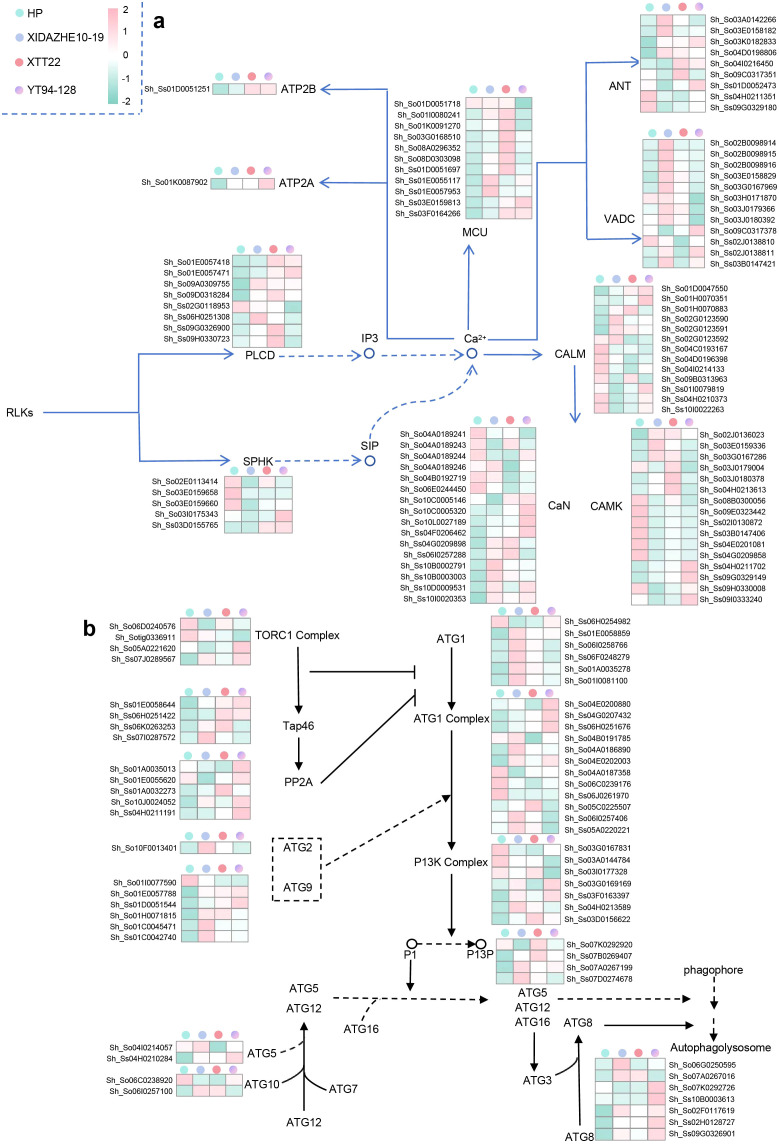
Expression dynamics of DEGs associated with calcium signaling and autophagy. **(a)** Calcium signaling cascade coupled with expression heatmaps of key gene families (e.g., PLCD, SPHK, MCU, CALM, and CAMK). **(b)** The autophagy pathway mapping the expression profiles of core ATG genes and complexes (TORC1, ATG1, PI3K, and the ATG8 conjugation system essential for autophagosome/autolysosome formation). In the heatmaps, rows represent individual genes and columns represent the four sugarcane cultivars (left to right: susceptible HP, resistant XIDAZHE10-19, susceptible XTT22, and resistant YT94-128, marked by top colored dots). The green (or cyan)-to-red color scale represents low-to-high relative expression levels. Solid arrows represent direct activation/interaction, dashed arrows denote indirect processes/translocation, and T-bars indicate inhibition.

To identify pivotal metabolites and subsequently pinpoint the key regulatory genes involved, we initially evaluated the global metabolomic variations across the different sugarcane cultivars. A comprehensive global metabolomic profiling successfully identified a total of 3,796 metabolites. Based on the metabolomic profiling results, metabolites with a Variable Importance in Projection (VIP) score > 1 were defined as differentially expressed metabolites (DEMs). In the YT94-128 vs. XTT22 comparison group, a total of 235 up-regulated and 219 down-regulated DEMs were identified. The YT94-128 vs. HP comparison group exhibited 307 up-regulated and 311 down-regulated DEMs. Furthermore, in the XIDAZHE10-19 vs. HP comparison, 335 up-regulated and 321 down-regulated DEMs were detected (**Figures 4a–c**). Finally, a Venn diagram was generated using the DEMs from these three comparison groups, revealing an intersection of exactly 69 shared metabolites across all three groups (**Figure 4h**; **Supplementary Table 6**).

**Figure 5:**
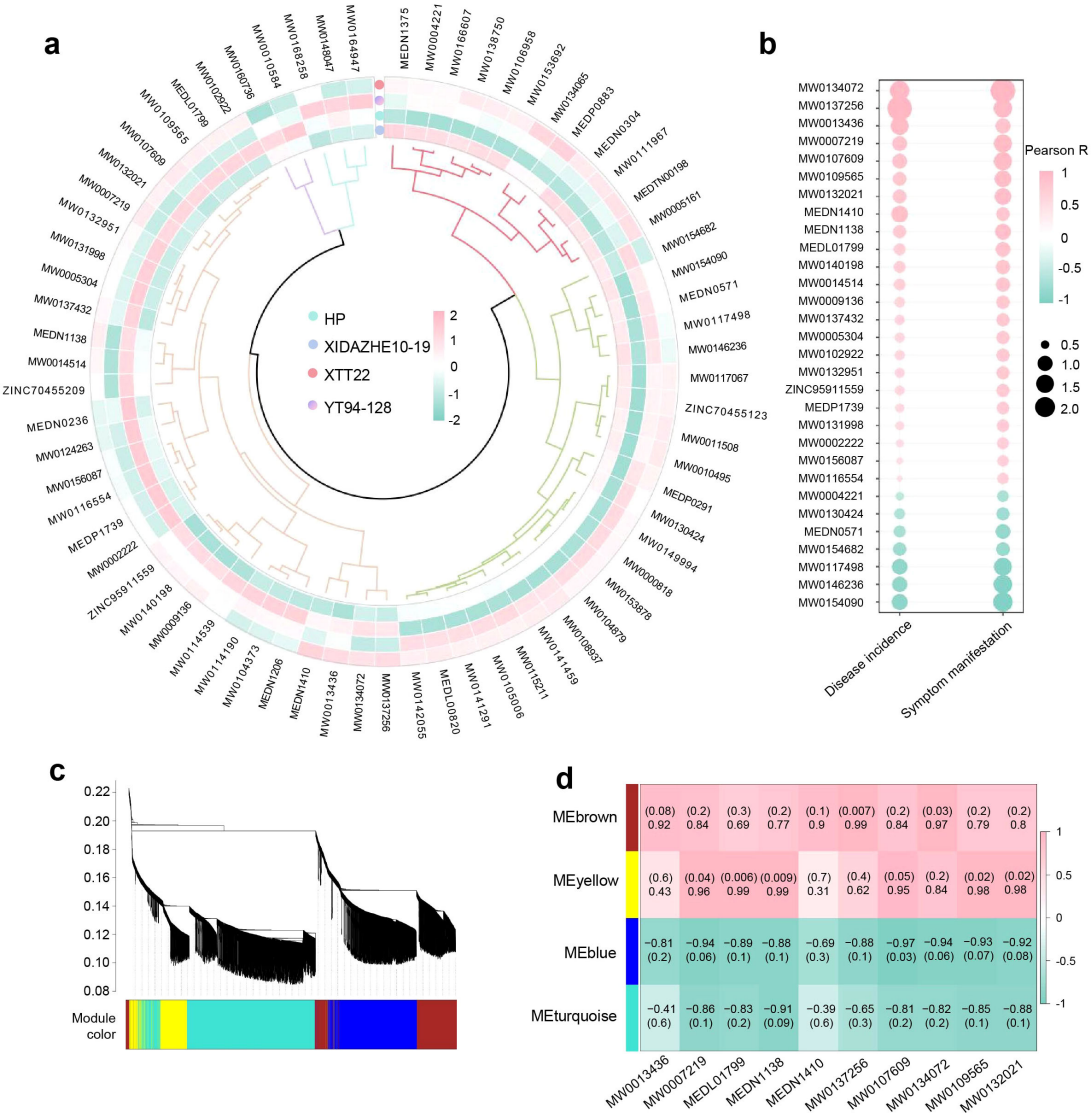
Expression patterns of differential metabolites, trait correlation, and Weighted Gene Co-expression Network Analysis (WGCNA). **(a)** Circular heatmap of abundance clustering for shared differential metabolites among three comparison groups (YT94-128 vs. HP, XIDAZHE10-19 vs. HP, and YT94-128 vs. XTT22). **(b)** Pearson correlation bubble plot of the top 30 differential metabolites most strongly correlated with two traits: “Disease incidence” and “Symptom manifestation”. The color indicates the direction and strength of the correlation (pink for positive correlation and cyan for negative correlation). **(c)** WGCNA hierarchical clustering dendrogram and module color assignment based on the common differentially expressed genes (DEGs) from the three comparison groups. **(d)** Correlation heatmap between WGCNA gene modules and the top 10 trait-associated metabolites. In each square, the upper value represents the correlation coefficient (pink for positive and cyan for negative), and the lower value in brackets represents the corresponding significance P-value.

Hierarchical clustering was performed on the abundances of differentially expressed metabolites (DEMs) across the different cultivars, and the results were visualized in a heatmap (**Figure 5a**). By integrating these metabolic profiles with mosaic disease incidence rates and typical symptoms, a correlation analysis was conducted to identify the top ten metabolites most highly associated with the disease (**Figure 5b**). From this highly correlated subset, multiple types of metabolites, broadly classified as lipid metabolism intermediates and cell membrane structural precursors (such as alcoholamines and glycerol derivatives), were identified (**Figures 5c–f**). These metabolites are closely related to disease resistance in both plants and animals (Dias et al., 2018; Shine et al., 2019; Wang et al., 2025c). Crucially, the results demonstrated a strict phenotype-associated accumulation pattern for these key metabolites: their abundances perfectly mirrored the disease susceptibility trend, being consistently highest in the highly susceptible cultivar HP, moderately accumulated in XTT22, and significantly suppressed in the resistant cultivars XIDAZHE10-19 and YT94-128.

To further the biological interpretation of these metabolic shifts and elucidate the potential molecular mechanisms underlying mosaic disease resistance, KEGG pathway enrichment analysis was performed on the differentially expressed metabolites (DEMs). In the YT94-128 vs. XTT22 comparison group, pathways related to plant disease resistance primarily included plant hormone signal transduction, tropane, piperidine and pyridine alkaloid biosynthesis, and phenylpropanoid biosynthesis. In the YT94-128 vs. HP comparison group, disease resistance-associated pathways involved the biosynthesis of various plant secondary metabolites, and phenylpropanoid biosynthesis. Furthermore, in the XIDAZHE10-19 vs. HP comparison group, the resistance-related pathways featured tropane, piperidine and pyridine alkaloid biosynthesis (**Figures 4d–f**). An intersection analysis across the three comparison groups revealed a total of 25 shared enriched pathways (**Figure 4g**) and 69 common differential metabolites (**Figure 4h**).

### 3.4 Integrated transcriptomic and metabolomic pathway analysis of sugarcane mosaic disease

To identify the key regulatory hub genes driving metabolic variations, weighted gene co-expression network analysis (WGCNA) was performed. Specifically, the abundances of the 10 key metabolites—which were strictly selected based on their highest correlation coefficients with mosaic disease incidence and typical symptom severity (**Figures 5a, b**)—were integrated with the 8,817 shared differentially expressed genes across the three comparison groups. Among them, the ‘yellow’ module was highlighted as the core regulatory module. As depicted in the module-trait relationship analysis (**Figure 5d**), the ‘yellow’ module exhibited the most robust and significant positive correlations with the vast majority of these key metabolites (with correlation coefficients r reaching up to 0.99). This strong positive association suggests that the genes clustered within this specific module are highly likely to act as positive regulators driving the biosynthesis or accumulation of these disease-responsive compounds. Consequently, 1,058 hub genes were ultimately screened from this core module for downstream interaction and functional analyses.

Hierarchical clustering was subsequently performed on these WGCNA-identified genes to generate an expression heatmap (**Supplementary Figure 3a**). The heatmap divided the genes into two major clusters based on their expression patterns. The first cluster exhibited the highest expression in HP, followed by XTT22, with lower expression levels in the two resistant cultivars. Conversely, the second cluster displayed relatively high expression in XIDAZHE10-19 and YT94-128, but lower expression in the two susceptible cultivars. Ultimately, based on these comprehensive expression profiles, *Sh_So02F0117494*—a gene belonging to the NDK family—was specifically highlighted due to its robust high expression in the two susceptible cultivars. Nucleoside diphosphate kinases (NDKs) are known to play crucial roles in cellular energy homeostasis and reactive oxygen species (ROS) signaling. Given its distinct phenotype-associated expression pattern, the significant up-regulation of *ShNDK* in susceptible cultivars suggests a potential association with the observed metabolic variations or general oxidative stress responses following viral infection. However, whether this specific expression profile acts as a primary driver of viral susceptibility or merely reflects a secondary consequence of metabolic disruption requires further experimental verification. The precise functional mechanisms of *ShNDK* during sugarcane mosaic disease remain to be fully elucidated.

**Figure 6:**
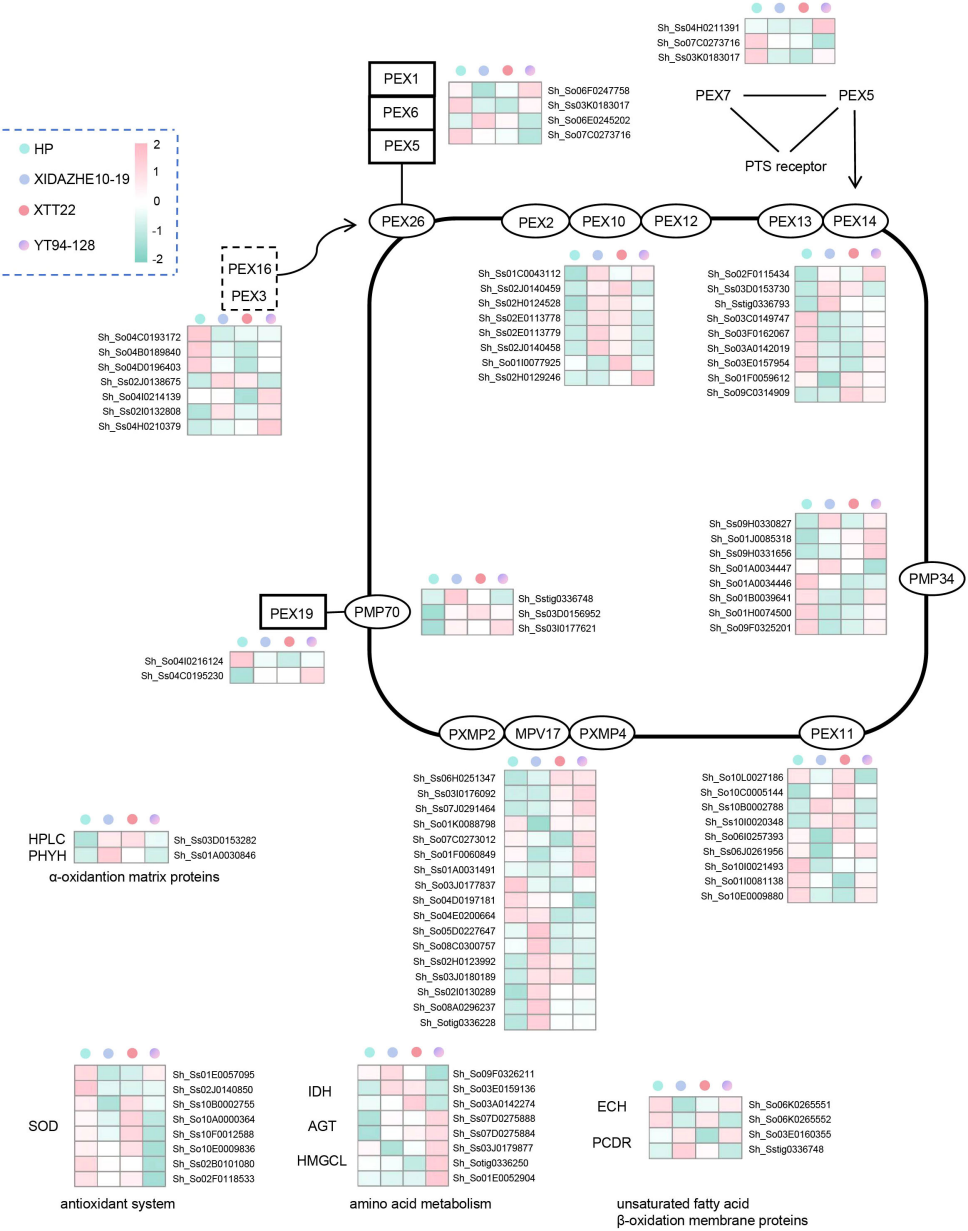
Expression dynamics of differentially expressed genes (DEGs) in the peroxisome pathway. Rows and columns represent individual genes and the four sugarcane cultivars, respectively (from left to right: susceptible HP, resistant XIDAZHE10-19, susceptible XTT22, and resistant YT94-128, marked by colored dots). The green-to-red color scale indicates low-to-high expression. Solid black arrows denote direct metabolic conversions or interactions.

The peroxisome pathway was identified among the shared pathways across the three transcriptomic comparison groups. By analyzing this pathway, we aimed to elucidate the differential expression profiles within the peroxisome pathway among the various cultivars (**Figure 6**). Furthermore, by integrating these findings with the expression dynamics of the *ShNDK* gene, we sought to infer the potential impact of this gene on the peroxisome pathway across different cultivars.

As illustrated, genes encoding the inner membrane channel protein MPV17, such as *Sh_So01F0060849*, exhibited relatively high expression in HP and YT94-128, whereas their expression levels were lower in XTT22 and XIDAZHE10-19 (**Figure 6**). In contrast, genes encoding superoxide dismutase 1 (SOD1), such as *Sh_Ss01E0057095*, displayed relatively higher expression levels in XIDAZHE10-19 and YT94-128 (**Supplementary Table 7**).

In conclusion, HP and YT94-128 maintained relatively high transcript abundances for organelle-related activities, including protein import and substance transport across the peroxisomal membrane. Conversely, these expression levels were notably lower in XTT22 and XIDAZHE10-19. Thus, it is reasonable to postulate that the disease resistance mechanisms of these latter two cultivars do not predominantly rely on the peroxisome pathway.

**Figure 7:**
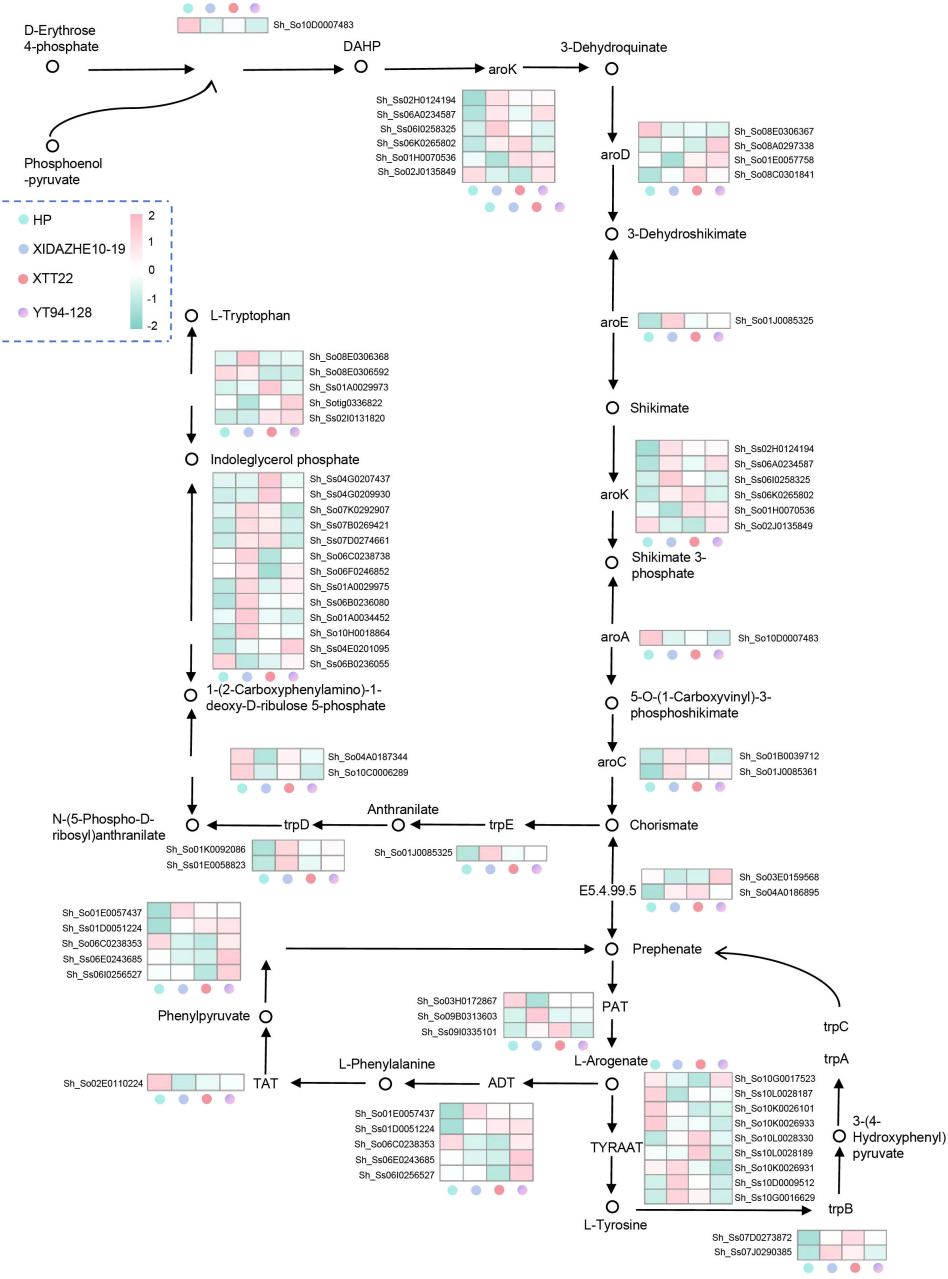
Expression dynamics of differentially expressed genes (DEGs) in the aromatic amino acid biosynthetic pathway. Rows and columns represent individual genes and the four sugarcane cultivars, respectively (from left to right: susceptible HP, resistant XIDAZHE10-19, susceptible XTT22, and resistant YT94-128, marked by colored dots). The green-to-red color scale indicates low-to-high expression. Solid black arrows denote direct metabolic conversions or interactions.

The phenylalanine, tyrosine, and tryptophan biosynthesis pathway was identified as a shared pathway across the three transcriptomic comparison groups (**Figure 7**). As illustrated, genes encoding tyrosine aminotransferase (TAT), such as *Sh_So02E0110224*—which catalyzes the transamination between aromatic amino acids and α-ketoglutarate—were highly expressed in susceptible cultivars but exhibited relatively lower expression levels in the others. Additionally, genes encoding chorismate mutase (EC 5.4.99.5), such as *Sh_So03E0159568*, along with those encoding shikimate kinase (aroK), a key enzyme in the shikimate pathway, such as *Sh_So02J0135849*, demonstrated higher expression levels in HP and YT94-128, whereas they were maintained at lower levels in XTT22 and XIDAZHE10-19 (**Supplementary Table 7**).

In conclusion, XIDAZHE10-19 exhibited relatively active expression profiles in the phenylalanine, tyrosine, and tryptophan biosynthesis pathway. It is reasonable to postulate that this cultivar preferentially utilizes this pathway to synthesize aromatic amino acids, thereby achieving enhanced disease resistance and stress tolerance.

### 3.5 RT-qPCR validation of sugarcane mosaic disease-associated genes

**Figure 8:**
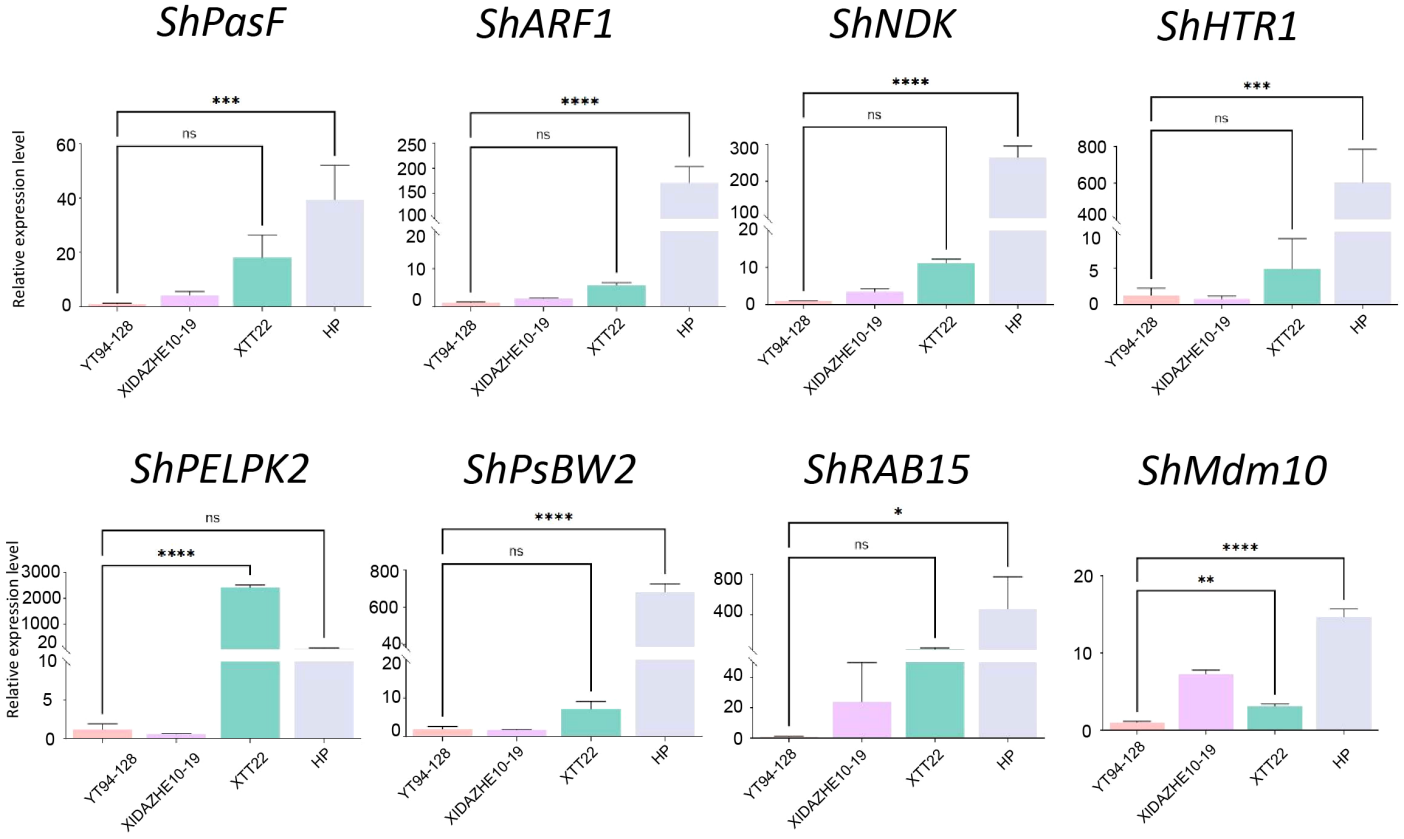
RT-qPCR validation of eight representative differentially expressed genes (DEGs) in sugarcane. To verify the reliability of the RNA-seq data, eight genes (*ShPasF*, *ShARF1*, *ShNDK*, *ShHTR1*, *ShPELPK2*, *ShPsBW2*, *ShRAB15*, and *ShMdm10*) were selected for RT-qPCR analysis across four samples (YT94-128, XIDAZHE10-19, XTT22, and HP). The Y-axis represents the relative expression level normalized to the GAPDH using the 2^−ΔΔCt^ method. Error bars indicate the standard deviation (SD) of three biological replicates. Asterisks indicate statistical significance: *P < 0.05, **P < 0.01, ***P < 0.001. while 'ns' indicates not significant (P > 0.05).

To verify the reliability of the experimental data, shared DEGs across the three comparison groups were selected for RT-qPCR validation. A total of eight genes were chosen, including the fatty acid desaturase gene *ShPasF7*, the ADP-ribosylation factor gene *ShARF1*, the core gene *ShNDK*, the histone H3 gene *ShHTR1*, the hydroxyproline-rich glycoprotein gene *ShPELPK2*, the photosynthetic electron transport gene *ShPsBW2*, the abscisic acid-responsive gene *ShRAB15*, and the mitochondrial distribution and morphology protein gene *ShMdm10*. The quantitative real-time PCR results demonstrated that the overall trends in relative expression levels (**Figure 8**) were highly consistent with the transcriptomic TPM values (**Supplementary Figure 4**), thereby confirming the reliability of the transcriptome sequencing results.

### 3.6 Protein-protein interaction and 3D structure prediction of the SCMD-associated *ShNDK*

**Figure 9:**
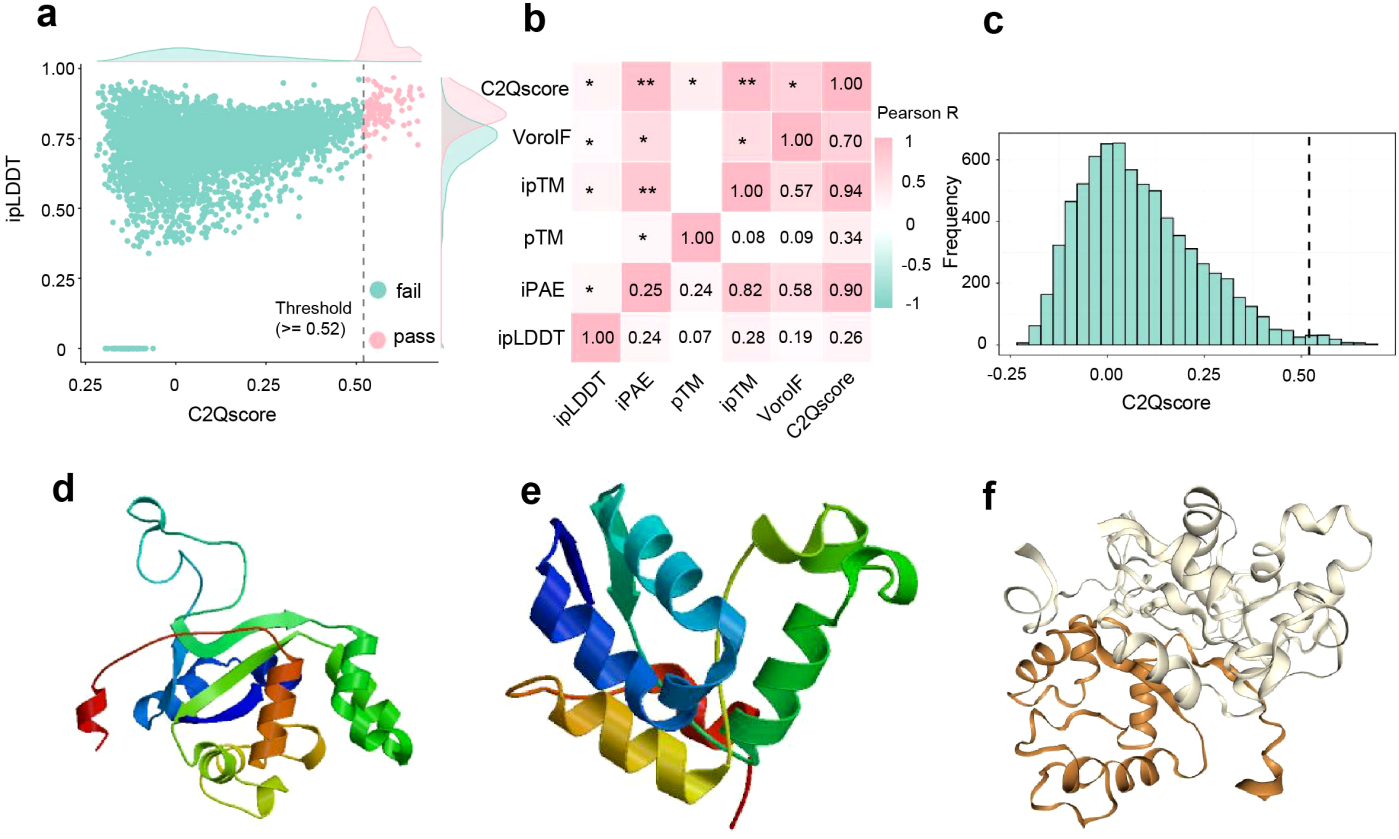
Quality evaluation of AlphaFold predictions and screening for high-confidence interaction targets. **(a)** Bivariate scatter and marginal density plots of ipLDDT vs. C2Qscore. The gray dashed line denotes the threshold (C2Qscore = 0.52). Cyan and pink dots represent substandard and selected high-quality models, respectively. **(b)** Pearson correlation heatmap of six core AlphaFold metrics. Colors indicate correlation strength (cyan: negative; white: none; pink: positive). Values in squares are Pearson coefficients (*P < 0.05, **P < 0.01). **(c)** C2Qscore distribution histogram of all 7,436 candidate models. The black dashed line indicates the stringent threshold (C2Qscore = 0.52) applied for identifying genuine interactions. **(d)** An NDK1 subfamily protein identified via AlphaFold-Multimer exhibiting interaction potential with the key gene product. **(e)** Predicted structure of the NDK1 protein encoded by *Sh_So02F0117494.* **(f)** Representative 3D architecture of the predicted protein-protein interaction complex. The model illustrates the stable oligomeric assembly of the target proteins, with critical interacting residues and specific structural domains at the binding interfaces visually highlighted to elucidate the precise interaction mechanisms.

To rigorously assess the reliability of the predicted protein-protein interaction interfaces for the core hub gene *ShNDK*, a comprehensive structural evaluation was performed. The complex topologies were initially modeled utilizing AlphaFold and HDOCK. Applying a stringent threshold of C2Qscore ≥ 0.52 (**Figure 8c**), the frequency distribution of the global prediction models exhibited a distinct right-skewed pattern. This distribution indicates that low-confidence interaction scores were effectively filtered out, while models exceeding the threshold retained high computational confidence. Furthermore, the scatter plot (**Figure 9a**) demonstrated that models satisfying this C2Qscore criterion also exhibited interface predicted local distance difference test (ipLDDT) scores highly concentrated in the upper tier. This confirms that the applied threshold successfully discriminates high-confidence structural models from background computational noise (represented by the green dots, indicating the fail group).

To cross-validate the robustness of this structural evaluation framework, a Pearson correlation analysis was performed across several established complex quality assessment metrics, including ipLDDT, iPAE, pTM, ipTM, VoroIF, and C2Qscore (**Figure 9b**). The structural scoring metrics exhibited a highly significant positive correlation (p < 0.01); specifically, the C2Qscore strongly correlated with the interface predicted template modeling score (ipTM, R = 0.94) and the interface predicted aligned error (iPAE, R = 0.90), as well as VoroIF (R = 0.70). This high degree of inter-metric consistency mathematically confirms that the C2Qscore serves as a reliable computational indicator for the structural quality and topological accuracy of the predicted interaction interfaces.

Under these stringent structural filtering criteria, 92 candidate proteins were identified, 17 of which were successfully annotated against the Swiss-Prot database, including NDK-type kinases and bZIP-type transcription factors (**Supplementary Table 9**). Given that proteins within the NDK family are structurally known to assemble into multimeric complexes (Lascu and Gonin, 2000). we focused our 3D structural modeling efforts on the allelic variants of the core gene to evaluate their assembly potential (**Figures 9d, e**). To strictly assess the topological fit and interaction energies of potential homodimeric or heterodimeric alignments, protein-protein docking analysis was performed utilizing the HDOCK server (**Figure 9f**; **Supplementary Table 10**). Among the predicted conformations, the heterodimer complex yielded the most optimal docking metrics. Specifically, with a ligand RMSD of 0.43 Å and a high docking confidence score of 0.9805, this specific structural alignment is computationally highly reliable, providing robust metric-driven support for the predicted physical assembly of these two variant proteins.

## 4 Discussion

A multi-year investigation preliminarily concludes that minimizing the use of diseased seed canes from the previous year can effectively mitigate the disease incidence in susceptible cultivars such as XTT22 (**Supplementary Tables 1**, **2**). However, for highly susceptible cultivars like HP, this practice exerts a negligible effect on disease incidence, regardless of whether newly planted or ratoon canes are cultivated. Routine field management and conventional breeding strategies are insufficient to restrict the transmission of sugarcane mosaic disease in cultivars like HP. Previous epidemiological investigations have demonstrated that the prevalent infection pattern of mosaic viruses in field-grown sugarcane consists of mixed infections, with double infection being the most common (He et al., 2022). Modern breeding strategies for sugarcane disease resistance have shifted comprehensively toward the development of broad-spectrum resistant cultivars and the mining of broad-spectrum resistance genes (Li et al., 2020; Lu et al., 2021). In our study, the cultivars YT94-128 and XIDAZHE10-19 all exhibited co-infection with SCMV and SCSMV, whereas HP and XTT22 presented a co-infection with SCMV and SrMV. The observation of a 100% co-infection rate among the sampled cultivars suggests that, under natural field stress, these plants had already activated systemic defense networks to cope with multiple viral infections (Suzuki et al., 2014). Consequently, the core hub genes identified from these co-infected samples via an integrated multi-omics (transcriptomics and metabolomics) approach are highly likely to be involved in the regulation of broad-spectrum resistance pathways.

Integrating the transcriptomic and pathway enrichment profiles of the resistant cultivars reveals a distinct divergence in core defense systems across different cultivars. This variation likely stems from their distinct genetic backgrounds and breeding histories, which have equipped them with divergent evolutionary adaptations and non-overlapping resistance (R) gene reservoirs to combat viral stress. XIDAZHE10-19 appears to employ a “containment and biochemical barrier” strategy, preferentially utilizing autophagy for the targeted degradation of invading viral components while concurrently channeling aromatic amino acids to fuel the massive biosynthesis of protective secondary metabolites (Ismayil et al., 2020; Maeda and Dudareva, 2012). However, it is necessary to critically consider alternative explanations for this metabolite accumulation. Rather than being solely an active defense mechanism, the buildup of certain metabolites could partially represent viral hijacking—where the virus actively reprograms host metabolism to facilitate its own replication—or merely serve as a byproduct of cellular damage and stress. Conversely, the defense response in YT94-128 is intimately associated with calcium signaling and the peroxisome pathway, indicating an “early signaling and oxidative burst” strategy. This effectively integrates our multi-omics findings: the activation of calcium and ROS signaling networks (observed in the transcriptome) directly drives the dynamic physiological shifts observed in our metabolomic profiling, such as the accumulation of glycerol derivatives and specific lipids that serve to mitigate oxidative damage (Aldon et al., 2018; Sandalio et al., 2021). Intriguing scientific questions thus emerge: Does XIDAZHE10-19 directly rely on robust, global transcriptional reprogramming to hyper-accumulate defense compounds, thereby overriding the sugarcane mosaic virus? Furthermore, does the activation of the calcium sensory network in YT94-128 further orchestrate downstream crosstalk between autophagy and ROS-mediated defense mechanisms? Ultimately, these compelling, multi-omics-derived hypotheses warrant further functional validation through subsequent molecular biological investigations.

To identify the upper-stream molecular switches that coordinate these intertwined pathways—particularly the balance between ROS generation and scavenging—we turned our focus to the key hub gene identified via WGCNA (**Figure 5c**), which belongs to the nucleoside diphosphate kinase (NDK) family. The autophagy pathway itself represents a highly compelling research frontier. Autophagosomes not only mediate the clearance of senescent and pathological endogenous metabolites, but also actively execute the targeted degradation of invading viral pathogens. Notably, alongside these canonical targets, autophagy also facilitates the scavenging of reactive oxygen species (ROS)—a function functionally analogous to the peroxisome pathway. Crucially, we found that autophagy−peroxisome crosstalk acts as the pivotal cellular link bridging these two discrete pathways. Recent studies have increasingly drawn attention to pexophagy, a highly selective form of autophagy, which has rapidly emerged as a frontline research direction in plant disease resistance (Calero-Muñoz et al., 2019; Demers et al., 2023; Kim et al., 2026; Wilhelm et al., 2022). This process specifically targets and eliminates dysfunctional, ROS-leaking peroxisomes, thereby eradicating potential upstream sources of oxidative stress. In doing so, autophagy−peroxisome crosstalk acts as a robust fail-safe mechanism for the peroxisomal network. Taken together, we postulate that a profound, synergistic crosstalk exists between the autophagy and peroxisome pathways in orchestrating plant immunity against viral infections.

During plant immune responses, the NDK gene family plays a pivotal role in activating the MAPK signaling cascades, which are intimately associated with disease resistance. Previous studies have elucidated that upon pathogen infection or stress induction, NDK specifically interacts with and activates MPK3/MPK6 within the MAPK cascade, thereby triggering the rapid phosphorylation and activation of a multitude of downstream disease-resistance transcription factors (Moon et al., 2003). In the present study, we discovered that the *ShNDK* gene exhibited remarkably high expression levels in the susceptible cultivars XTT22 and HP, While paradoxical, this phenomenon is frequently observed in plant-pathogen interactions: highly susceptible plants often launch a delayed, hyperactive transcriptional burst—a “futile cycle” triggered by massive viral replication—that ultimately fails to mount a timely physiological barrier. We hypothesize that *ShNDK* functions as a pivotal positive regulator of plant defense. Its pronounced upregulation in susceptible cultivars is hypothesized to represent a passive, compensatory stress response triggered by massive viral replication; however, due to the potential lack of necessary downstream cofactors—such as specific transcription factors or coordinated autophagic machinery—this robust expression might ultimately be insufficient to rescue the susceptible phenotype. Furthermore, genes of the NDK family are implicated in regulating the biosynthesis of peroxides, glycerophospholipids, and diverse lipids—metabolic classes that are intricately linked to physiological defense mechanisms underlying plant stress tolerance and disease resistance (Dorion and Rivoal, 2018; Lim et al., 2017). Consequently, we postulate that the *ShNDK* gene orchestrates sugarcane’s physiological disease resistance processes by dynamically modulating these defense-associated metabolic networks.

By integrating 3D structure prediction with AlphaFold analysis, in addition to the proteins previously delineated in the results section, we successfully identified other NDK family members, such as NDK1 and NDK4, as potential interactors (**Supplementary Table 9**). Our structural analysis directly aligns with the established biochemical paradigms of the NDK family. Crucially, members of this conserved kinase family are well-documented to engage in self-interaction or homotypic assembly to form functional homo- or hetero-oligomers (such as hexamers or dimers), which is chemically indispensable for augmenting and sustaining their full catalytic activity (Lascu and Gonin, 2000), In perfect agreement with this classical framework, our HDOCK and AlphaFold-Multimer simulations successfully captured this structural propensity in sugarcane variants, specifically yielding a highly reliable heterodimeric interface; Subsequently, the functionally active NDK oligomers may further translocate to peroxisomes or directly bind to the core enzymes within them (such as catalase). Through this mechanism, they potentially fine-tune the reactive oxygen species (ROS) defense machinery to confer robust disease resistance (Fukamatsu et al., 2003). Moving forward, we intend to clone this candidate gene to generate overexpression lines and mutant plants in both rice and sugarcane. By subjecting these genetically modified plants to mosaic virus inoculation assays, we will systematically investigate their resistance phenotypes.

Among the candidate NDK-interacting proteins identified through AlphaFold-mediated screening, we observed that several candidates belong to canonical disease-resistance transcriptional regulatory families, encompassing bZIP, Dof, and zinc finger proteins (ZFPs) (**Supplementary Table 9**). These transcription factors serve as central hubs in orchestrating plant defense networks (Alves et al., 2013; Kiełbowicz-Matuk, 2012). Beyond protein-coding hub genes such as *ShNDK*, long non-coding RNAs (lncRNAs) have emerged as critical modulators of plant defense, as demonstrated in cotton where *lncRNA7* and *lncRNA2* regulate cell wall-associated genes to confer Verticillium wilt resistance (Zhang et al., 2022). Future investigations in sugarcane should explore whether analogous *lncRNA* regulatory nodes operate alongside *ShNDK* to fine-tune mosaic virus resistance. Furthermore, the candidate interactome encompasses pathogenesis-related (PR) proteins directly implicated in disease progression (Datta et al., 1999), alongside specific proteins involved in epigenetic modifications (Feng et al., 2019; Tada et al., 2008). Taken together, we postulate that NDK physically associates with these defense-related transcription factors or their homologous counterparts, thereby synergistically potentiating the activation of plant defense networks at the protein level. However, direct experimental evidence substantiating this hypothesis is currently lacking. Future investigations will entail the generation of targeted transgenic lines. By coupling site-directed mutagenesis with co-immunoprecipitation (Co-IP) and Western blot (WB) analyses, we aim to systematically monitor alterations in the binding affinity of these protein complexes in the absence of the NDK protein or upon the mutation of critical interaction interfaces.

Furthermore, based on our findings and previous literature, we propose a conceptual model illustrating how the hub gene *ShNDK* orchestrates sugarcane defense against SCMD viral infection (**Supplementary Figure 5**) (Dorion and Rivoal, 2018; Lim et al., 2017). Upon mosaic virus perception, the initial influx of intracellular Ca^2+^ signals activates *ShNDK*, which subsequently boosts GTP production to switch downstream Rop/Rac small G-proteins into their active state. This active complex stimulates membrane-bound NADPH oxidase (RBOH) to trigger a reactive oxygen species (ROS) burst. Crucially, the peroxisome pathway serves as a vital regulatory node within this network, finely tuning the accumulation of H_2_O_2_ to maintain cellular redox homeostasis. Together, this *ShNDK*-mediated regulatory axis balances robust immune signaling with cellular protection during viral invasion. It is critical to note that this *ShNDK*-ROS-peroxisome-autophagy regulatory axis represents a preliminary working hypothesis. While strongly supported by integrated transcriptomic and metabolomic evidence, validating the precise causative links within this dynamic network will be the primary focus of our forthcoming molecular investigations.

## 5 Conclusion

In this study, through multi-dimensional omics analysis and field validation, we comprehensively elucidated the resistance differences among various sugarcane cultivars to sugarcane mosaic disease and their underlying molecular mechanisms. Field phenotypic identification clearly classified HP and XTT22 as susceptible, whereas XIDAZHE10-19 and YT94-128 exhibited stable resistance. Our key findings reveal that resistant cultivars effectively defend against viral infection primarily by activating the calcium signaling pathway and the autophagy-mediated defense network, as well as by enhancing the aromatic amino acid biosynthesis pathway (particularly in XIDAZHE10-19). Through transcriptomic and Weighted Gene Co-expression Network Analysis (WGCNA), we identified 8,817 shared DEGs and pinpointed an NDK family member, *ShNDK* (*Sh_So02F0117494*)—which is significantly highly expressed in susceptible cultivars—as the core responsive hub gene. Furthermore, spatial structural and interaction network analyses of the core protein *ShNDK* confirmed that its allelic variants can assemble into highly stable oligomeric conformations and physically interact with specific kinases and bZIP transcription factors.

Despite these comprehensive insights, this study has certain limitations. The protein-protein interactions and oligomeric conformations of *ShNDK* were primarily established through advanced computational predictions (e.g., AlphaFold) and require further *in vivo* and *in vitro* experimental validation, such as co-immunoprecipitation or yeast two-hybrid assays. Additionally, the direct functional efficacy of *ShNDK* in conferring viral resistance has yet to be verified through genetic transformation (e.g., overexpression or knockout) in planta.

Regarding future implications, *ShNDK* and the identified autophagy/peroxisome-associated pathways represent priority targets for marker-assisted selection or CRISPR-based resistance enhancement. In summary, this study not only systematically elucidates the multiple molecular regulatory networks involved in sugarcane defense against sugarcane mosaic disease but also provides crucial genetic targets and a solid theoretical foundation for cultivating new, highly resistant sugarcane cultivars through precision molecular breeding.

## Data Availability

The raw data supporting the conclusions of this article will be made available by the authors, without undue reservation.

## Supplementary material

Supplementary Figure 1 DEG expression patterns, sample correlations, and GO enrichment analysis. **(a)** Cluster heatmap of shared DEGs across three comparisons (XIDAZHE10-19 vs. HP, YT94-128 vs. HP, and YT94-128 vs. XTT22), with colors indicating normalized expression. **(b)** Pearson correlation heatmap of biological replicates. **(c–e)** Volcano plots for **(c)** YT94-128 vs. HP, **(d)** XIDAZHE10-19 vs. HP, and **(e)** YT94-128 vs. XTT22. Pink and cyan dots denote significantly up- and down-regulated genes, respectively. **(f, g)** GO enrichment bar charts of DEGs for **(f)** YT94-128 vs. HP and **(g)** YT94-128 vs. XTT22 (x-axis represents −Log10(P-value)).

Supplementary Figure 2 Clustering heatmaps of DEGs in key biological pathways. The heatmaps show the relative expression profiles of specific DEGs across four samples (HP, XTT22, XIDAZHE 10-19, and YT94-128). Colors indicate Z-score normalized expression (pink: high; cyan: low). Row dendrograms represent hierarchical clustering of genes based on expression similarity, and columns indicate samples. Expression heatmaps are shown for genes involved in **(a)** calcium signaling, **(b)** autophagy, **(c)** peroxisome biogenesis/function, and **(d)** phenylalanine metabolism.

Supplementary Figure 3 Expression heatmap of WGCNA hub genes. The heatmap displays the expression profiles of hub genes selected from key co-expression modules across four sugarcane cultivars. The color scale indicates Z-score normalized expression (pink: high; cyan: low). Top and left dendrograms show the hierarchical clustering of samples and genes, respectively.

Supplementary Figure 4 Expression levels of eight selected candidate genes based on RNA-seq data. The bar charts illustrate the transcript abundance of eight representative genes (*ShPasF*, *ShARF1*, *ShNDK*, *ShHTR1*, *ShPELPK2*, *ShPsBW2*, *ShRAB15*, and *ShMdm10*) across four sugarcane samples (YT94-128, XIDAZHE10-19, XTT22, and HP). The Y-axis represents the normalized expression level calculated as Transcripts Per Million (TPM). Error bars indicate the standard deviation (SD) of the biological replicates.

Supplementary Figure 5 Schematic model of the *ShNDK*-mediated regulation of the peroxisome pathway. Upon perception of upstream stress signals, *ShNDK* is activated to promote GTP production, which subsequently recruits and triggers Rop/Rac GTPases. The active Rop/Rac-GTP complex stimulates membrane-bound NADPH oxidase (RBOH) to modulate peroxisome-mediated homeostasis and coordinate downstream defense responses, including redox signaling, antioxidant enzyme activation, programmed cell death (PCD), and immune regulation. Black lines indicate extracellular or upstream perception processes; blue lines represent intracellular signaling pathways; green lines/arrows denote activation and promotion; red lines indicate negative regulation and inhibition.
