# Supplementary figures and images for: Integrated transcriptomic and metabolomic profiling reveals coordinated regulatory networks associated with mosaic disease resistance in sugarcane
Source: Front Plant Sci. 2026 Jul 20;17:1891189. doi: 10.3389/fpls.2026.1891189 (PMC13431650; doi:10.3389/fpls.2026.1891189)

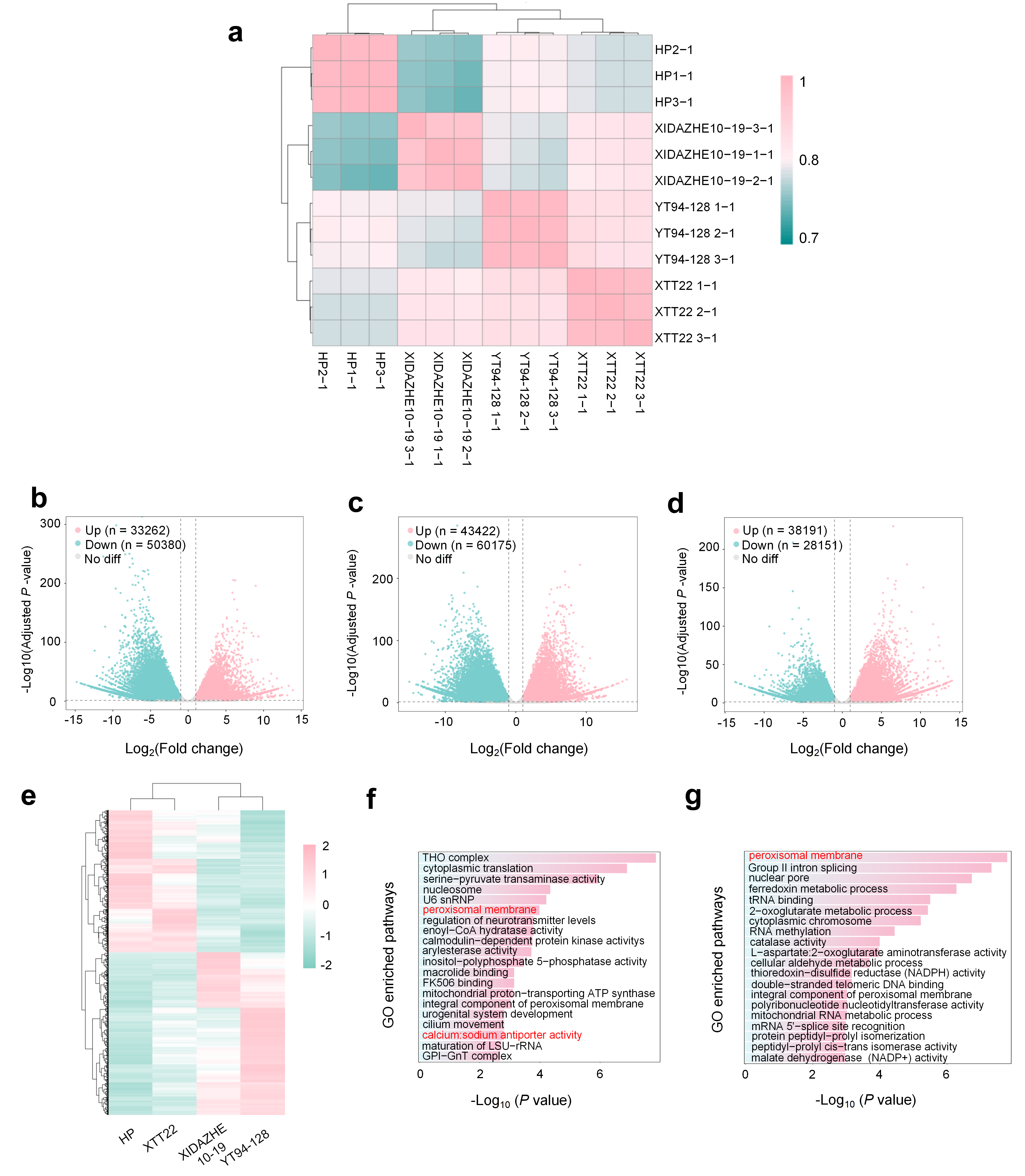

Supplement: Supplementary Figure 1 — DEG expression patterns, sample correlations, and GO enrichment analysis. (a) Cluster heatmap of shared DEGs across three comparisons (XIDAZHE10-19 vs. HP, YT94-128 vs. HP, and YT94-128 vs. XTT22), with colors indicating normalized expression. (b) Pearson correlation heatmap of biological replicates. (c–e) Volcano plots for (c) YT94-128 vs. HP, (d) XIDAZHE10-19 vs. HP, and (e) YT94-128 vs. XTT22. Pink and cyan dots denote significantly up- and down-regulated genes, respectively. (f, g) GO enrichment bar charts of DEGs for (f) YT94-128 vs. HP and (g) YT94-128 vs. XTT22 (x-axis represents −Log10(P-value)). [file Image1.png]

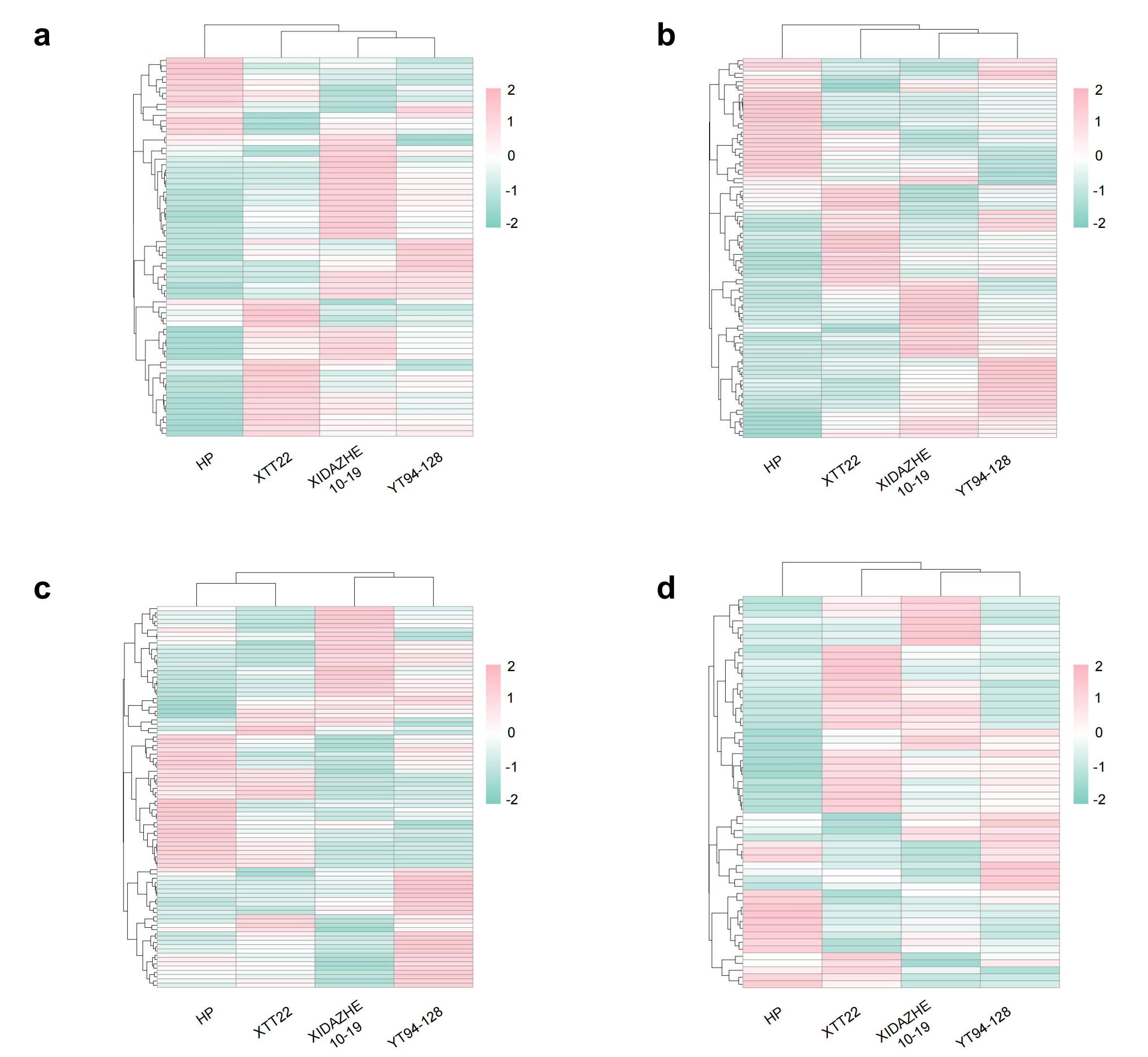

Supplement: Supplementary Figure 2 — Clustering heatmaps of DEGs in key biological pathways. The heatmaps show the relative expression profiles of specific DEGs across four samples (HP, XTT22, XIDAZHE 10-19, and YT94-128). Colors indicate Z-score normalized expression (pink: high; cyan: low). Row dendrograms represent hierarchical clustering of genes based on expression similarity, and columns indicate samples. Expression heatmaps are shown for genes involved in (a) calcium signaling, (b) autophagy, (c) peroxisome biogenesis/function, and (d) phenylalanine metabolism. [file Image2.png]

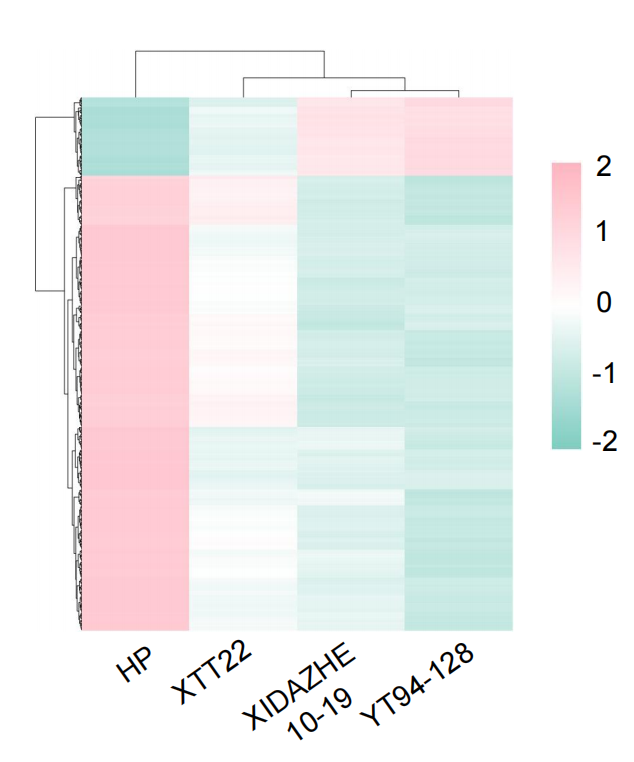

Supplement: Supplementary Figure 3 — Expression heatmap of WGCNA hub genes. The heatmap displays the expression profiles of hub genes selected from key co-expression modules across four sugarcane cultivars. The color scale indicates Z-score normalized expression (pink: high; cyan: low). Top and left dendrograms show the hierarchical clustering of samples and genes, respectively. [file Image3.png]

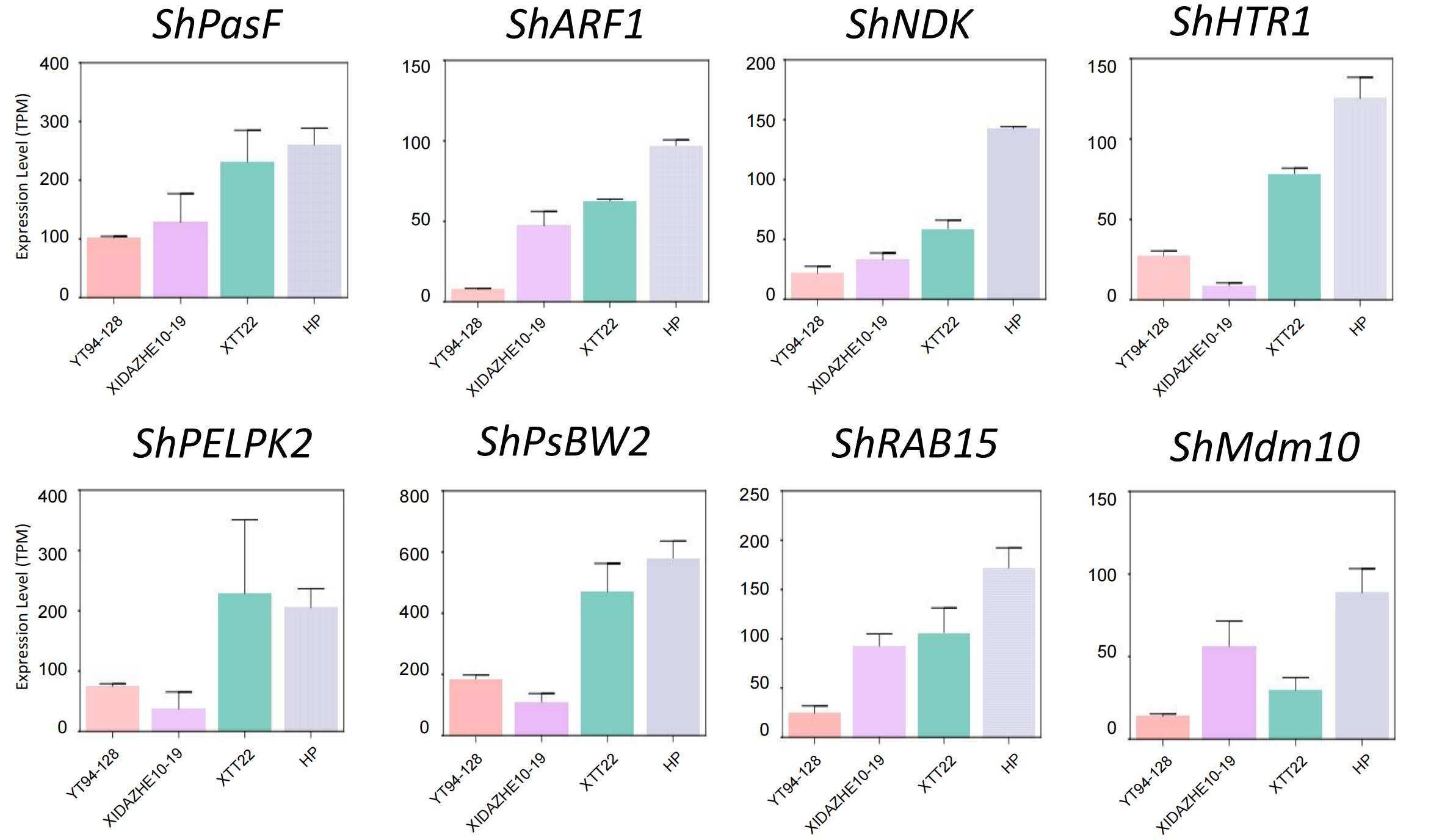

Supplement: Supplementary Figure 4 — Expression levels of eight selected candidate genes based on RNA-seq data. The bar charts illustrate the transcript abundance of eight representative genes (ShPasF, ShARF1, ShNDK, ShHTR1, ShPELPK2, ShPsBW2, ShRAB15, and ShMdm10) across four sugarcane samples (YT94-128, XIDAZHE10-19, XTT22, and HP). The Y-axis represents the normalized expression level calculated as Transcripts Per Million (TPM). Error bars indicate the standard deviation (SD) of the biological replicates. [file Image4.png]

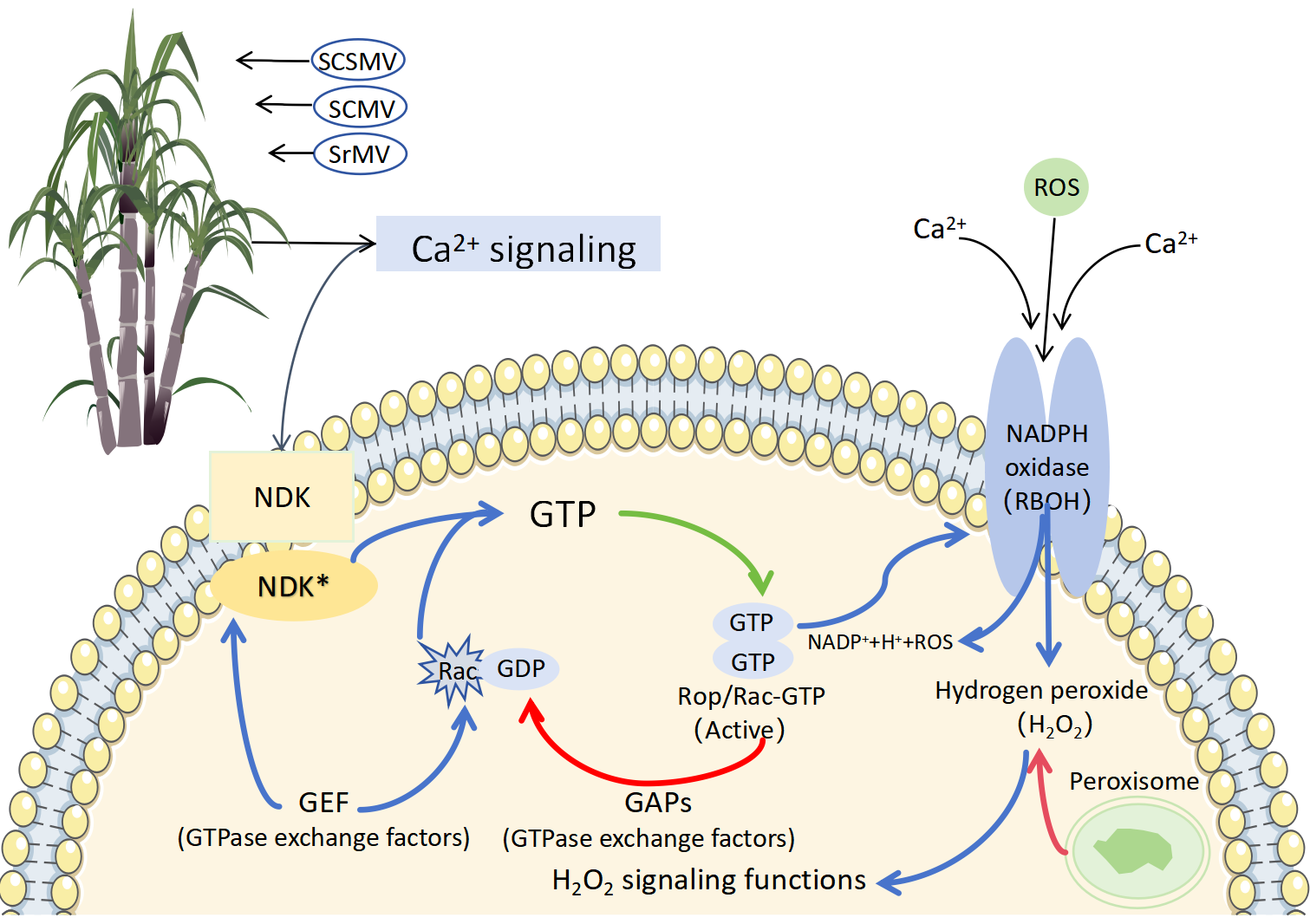

Supplement: Supplementary Figure 5 — Schematic model of the ShNDK-mediated regulation of the peroxisome pathway. Upon perception of upstream stress signals, ShNDK is activated to promote GTP production, which subsequently recruits and triggers Rop/Rac GTPases. The active Rop/Rac-GTP complex stimulates membrane-bound NADPH oxidase (RBOH) to modulate peroxisome-mediated homeostasis and coordinate downstream defense responses, including redox signaling, antioxidant enzyme activation, programmed cell death (PCD), and immune regulation. Black lines indicate extracellular or upstream perception processes; blue lines represent intracellular signaling pathways; green lines/arrows denote activation and promotion; red lines indicate negative regulation and inhibition. [file Image5.png]
